# Human antibody repertoire frequently includes antibodies to a bacterial biofilm associated protein

**DOI:** 10.1371/journal.pone.0219256

**Published:** 2019-07-09

**Authors:** Stefan Ryser, Edgar Tenorio, Angeles Estellés, Lawrence M. Kauvar

**Affiliations:** Trellis Bioscience LLC, Redwood City, California, United States of America; Universidade Nova de Lisboa, PORTUGAL

## Abstract

We have previously described a native human monoclonal antibody, TRL1068, that disrupts bacterial biofilms by extracting from the biofilm matrix key scaffolding proteins in the DNABII family, which are present in both gram positive and gram negative bacterial species. The antibiotic resistant sessile bacteria released from the biofilm then revert to the antibiotic sensitive planktonic state. Qualitative resensitization to antibiotics has been demonstrated in three rodent models of acute infections. We report here the surprising discovery that antibodies against the target family were found in all twenty healthy humans surveyed, albeit at a low level requiring a sensitive single B-cell assay for detection. We have cloned 21 such antibodies. Aside from TRL1068, only one (TRL1330) has all the biochemical properties believed necessary for pharmacological efficacy (broad spectrum epitope specificity and high affinity). We suggest that the other anti-DNABII antibodies, while not necessarily curative, reflect an immune response at some point in the donor’s history to these components of biofilms. Such an immune response could reflect exposure to bacterial reservoirs that have been previously described in chronic non-healing wounds, periodontal disease, chronic obstructive pulmonary disease, colorectal cancer, rheumatoid arthritis, and atherosclerotic artery explants. The detection of anti-DNABII antibodies in all twenty surveyed donors with no active infection suggests that bacterial biofilm reservoirs may be present periodically in most healthy individuals. Biofilms routinely shed bacteria, creating a continuous low level inflammatory stimulus. Since chronic subclinical inflammation is thought to contribute to most aging-related diseases, suppression of bacterial biofilm has potential value in delaying age-related pathology.

## Introduction

Many serious bacterial infections are difficult to treat due to formation of a biofilm matrix, which not only protects the bacterial cells from attack by the cellular immune system but also induces a physiological shift from the planktonic (free floating) to a slower growing sessile (adherent) state [1]. Antibiotic sensitivity differs between these two states, with biofilm associated bacteria typically showing as much as 1000-fold less sensitivity to antibiotics. The CDC and others estimate that 65–80% of clinically significant bacterial infections are drug refractory due to biofilm [2].

Biofilms have been found to include proteins and extracellular polyglycans from both bacteria and the host, as well as considerable amounts of extracellular DNA (eDNA), whose key structural role in the biofilm matrix was first established by showing that DNAse can disrupt biofilms [3]. In newly forming bacterial colonies, cooperative quorum sensing causes a stress response that includes explosive release of DNA from a small portion of the bacterial community [4]. This eDNA serves as a scaffold for the remaining biofilm components. Comparative proteomic studies have identified dozens of proteins characteristic of the sessile state [5]. Prominent among these is the DNABII family of DNA binding proteins, including Integration Host Factor (IHF) and Histone-like DNA-Binding Proteins (HU), both of which bind eDNA in a sequence non-specific manner [6]. The location of IHF in the biofilm matrix has been visualized by antibody labeling, revealing its localization at anchoring nodes in the matrix [7]. This sequence of events from micro-colony formation to established biofilm communities is widespread, including cooperative formation of biofilms by more than one bacterial species.

Such biofilm protected bacterial colonies can develop wherever small bacterial aggregates are able to form. Such reservoirs have been described in chronic non-healing wounds [8], persistent periodontal disease [9], chronic obstructive pulmonary disease (COPD) [10], and inflammatory bowel disease [11] among others. For example, bacterial biofilms were found in 89% of tumors in the ascending colon (n = 19) [12]. Further, procarcinogenic colon tissue inflammation (*e*.*g*. elevated IL-6) was associated with biofilm formation even in healthy subjects without colorectal cancer.

Another example comes from a study of atherosclerotic artery explants from 15 patients with advanced atherosclerosis [13]. All showed evidence of bacterial presence by PCR of bacterial ribosomal RNA, with *Pseudomonas aeruginosa* specifically identified in six of the samples along with lower frequencies of ten other bacterial species. In five of the samples, the length of artery segment was sufficient to allow histochemical staining of bacterial nucleic acid using a fluorescent probe. In all five, microcolonies were visualized comprising a few dozen to a few hundred detectable probe targets located proximal to the internal elastic lamina and associated with fibrous tissue.

Reservoirs of bacteria that do not induce symptoms of an acute infection such as fever and clinically observable inflammation are sometimes referred to as bacterial colonization rather than infection [14]. Nonetheless, they contribute to pathology. For example, dispersion of a biofilm of *Propionibacterium acnes* (also found in atherosclerotic plaques) has been observed *in vitro* in response to physiologically relevant levels of norepinephrine in the presence of transferrin [15]. This dispersion was accompanied by secretion of lytic enzymes with the potential to damage surrounding tissues. The same authors documented that lipases released by *P*. *acnes* act as a chemoattractant for human neutrophils *in vitro* which is significant in light of the demonstration that neutrophils play a major role in plaque formation in mice genetically deficient for LDL receptor [16]. Thus, the host response to biofilm likely contributes to plaque formation.

Dispersion is an important feature of biofilms in general as it facilitates infection of secondary sites. Experimentally, most of the cells produced in a biofilm will eventually detach and enter the aqueous phase [17]. Mathematical modeling of the dynamic biofilm structure suggests that coupling the dispersal rate to quorum sensing enables maturation of the biofilm community before shifting to a mode of shedding cells [18]. In combination, the behaviors of bacteria in biofilms support an ecology in the body of cryptic colonies that could provoke localized inflammatory reactions, even in the absence of fulminating infection.

TRL1068 is a native human monoclonal antibody (mAb) that binds with high affinity to a conformational epitope on DNABII proteins that is conserved across both gram positive and gram negative bacteria [19]. This epitope is far more conserved than the rest of the protein, indicative of a critical function. *In vitro*, TRL1068 acts by binding DNABII proteins, thereby shifting the binding equilibrium of these proteins from the biofilm bound state towards the free state thereby eliminating the nodes needed to achieve the three-dimensional meshwork structure [20]. The resulting biofilm disruption is accompanied by release of bacteria that revert to the antibiotic sensitive planktonic state.

TRL1068 has shown *in vivo* efficacy for treatment of acute biofilm infections in three animal models using antibiotic resistant strains: infected implants and infective endocarditis with *Staphylococcus aureus* [19], and infected catheter in soft tissue with A*cinetobacter baumannii* [21]. Highly significant potentiation of conventional bactericidal drugs has thereby been shown for daptomycin, vancomycin and imipenem.

TRL1068 was cloned by using a proprietary single B-cell mAb discovery technology [22]. Approximately 20 million B-cells were screened, drawn from 20 anonymized healthy human blood donors. In addition to TRL1068, 20 other mAbs were cloned. The properties of these additional mAbs are described here, and the potential implications for chronic inflammatory diseases are discussed.

## Materials and methods

### Antigen production: Integration Host Factor (IHF) and Histone-like DNA-Binding Proteins (HU)

IHF and HU proteins from *Staphylococcus aureus*, *Pseudomonas aeruginosa*, *Klebsiella pneumoniae*, *Haemophilus influenzae*, *Acinetobacter baumannii* were produced as 6xHis-tagged proteins by transient transfection in HEK293 Freestyle cells (Thermo Fisher Scientific, Waltham, MA) as previously described [19].

### IHF and HU peptides

As previously described [19], epitope mapping was accomplished by measuring mAb binding against a set of 26 linear peptides comprising overlapping 15-mers spanning the entire *S*. *aureus* HU protein, with offsets of 3 residues. These biotinylated peptides were synthesized by Mimotopes Pty. Ltd. (Victoria, Australia).

### Single B-cell mAb discovery technology

As previously described [19], leukopaks were obtained from a total of 20 anonymized donors under informed consent approved by Stanford’s Institutional Review Board #5136 panel 7 (Stanford Blood Center; Stanford, CA): protocol ID 13942. Peripheral Blood Mononuclear Cells (PBMCs) were prepared by standard methods and individual memory B-cells assayed following stimulation to proliferate and differentiate into plasma cells [22]. A portion of the cells was allowed to settle to the surface and secrete IgG with these single cell footprints screened using a multiparameter assay that allows concurrent measurement of binding to different targets conjugated to distinguishable fluorescent beads. Three recombinant DNABII proteins with diverse sequences were used for the primary screen: HU from *S*. *aureus*, IHF from *K*. *pneumoniae*, and IHF from *H*. *influenzae* with a fourth bead type coated with BSA as a control for non-specific binding. Since the percentage of memory B-cells (CD19/CD27 positive cells) in PBMCs varied among different blood donors (0.4–4.0%), the number of memory B-cells tested in a single experiment was normalized to ~200,000 memory B-cells. After identifying a B-cell secreting a mAb meeting the selection criteria, the encoding mRNAs for heavy and light chains were amplified by single cell RT-PCR from sibling cells and cloned into an expression vector for transfection in HEK293 Freestyle cells (Thermo Fisher Scientific, Waltham, MA).

### ELISA for full length IHF and HU proteins

Specific binding was evaluated against purified IHF/HU proteins from *S*. *aureus*, *P*. *aeruginosa*, *K*. *pneumoniae*, *H*. *influenzae* and *A*. *baumannii* in an ELISA assay as previously described [19]. The 6xHis-tagged antigens were purified using His60 beads (Clontech Laboratories, Mountain View, CA) according to the manufacturer’s recommendations. Antibodies were purified using immobilized goat anti-human Fc-specific antibody (Jackson ImmunoResearch Laboratories, West Grove, PA). Proteins were passively adsorbed on a Microlon High Binding plate (Grenier/Thermo Fisher Scientific, Waltham, MA) overnight at 4°C and the mAbs diluted in blocking buffer (PBS/3% BSA) for addition to the plate in serial dilutions. Horseradish peroxidase (HRP)-conjugated anti human IgG was used as detection antibody in conjunction with TMB (tetramethylbenzidine) substrate, recording optical density at 450 nm. Affinity calculations (mid-point of the ELISA binding curve) were performed using a 4-parameter logistic regression curve fit add-in to Microsoft Excel.

### ELISA for peptide mapping

Biotinylated peptides were screened by an ELISA assay similar to that for the full-length proteins except that Pierce Streptavidin Coated High Sensitivity Plates (Thermo Fisher Scientific, Waltham, MA) were used following manufacturer recommendations. The biotinylated peptides (26 linear peptides comprising overlapping 15-mers spanning the entire *S*. *aureus* HU protein, with offsets of 3 residues) were diluted in DMSO (15–25 mg/mL, 5 mM), and further diluted up to 1:1000 in 0.1% BSA.

## Results

Memory B-cells from 20 randomly selected healthy adult donors to the Stanford Blood Center were screened using the previously described single B-cell assay (CellSpot) [19]. Hits in the primary screening assay were recorded for all 20, with frequency varying between 1.7 and 10.8 memory cells per 100,000 B-cells surveyed. Table 1 summarizes the qualitative binding specificity of the 21 mAbs cloned from eight of these blood samples in a single point screening ELISA measuring binding from cell culture supernatants to a panel of four DNABII homologs from bacterial species displaying maximal variation in the sequence across the DNABII family (*S*. *aureus*; *P*. *aeruginosa*; *K*. *pneumoniae*; *H*. *influenzae*). The values were internally consistent across assays, but were determined on different days for different samples; although still useful for specificity assessment, we regard the values as only semi-quantitative with regard to absolute affinity, particularly since a more direct assay of affinity, *e*.*g*. by biosensor analysis of on and off rates, was not conducted. Also shown are the germ line origins of the VH and VL segments. Table 2 summarizes the affinities of the strongest binding mAbs from Table 1, as estimated using the point of inflection of 4-paramter logistic curve fit to the ELISA binding curves, assayed using mAbs purified by binding to immobilized goat anti-human Fc-specific antibody; see Supplemental Information for the underlying data.

**Table 1:** Anti-DNABII mAbs.

mAb TRL#	PBMC Donor	ELISA OD yellow = binding white = non-binding (below BSA background of 0.06)				Heavy Chain Variable Region Germline	Light Chain Variable Region Germline	
		*S*.*a*. HU beta	*P*.*a*. IHF alpha	*K*.*p*. IHF alpha	*H*.*i*. IHF alpha			
1068	SBC230	1.55	1.6	1.6	1.6	IGHV4-59*01	IGKV3-20*01	Kappa
1330	SBC248	3.0	2.9	2.8	3.0	IGHV1-18*01	IGLV2-8*01	Lambda
1215	SBC248	1.4	1.7	1.6	1.9	IGHV1-18*01	IGLV2-8*01	Lambda
1216	SBC248	0.5	0.7	0.4	0.06	IGHV3-30*03	IGLV3-10*01	Lambda
1261	SBC236	0.5	0.7	0.4	0.06	IGHV3-33*01	IGKV4-1*01	Kappa
1262	SBC236	1.5	0.9	1.4	0	IGHV3-33*01	IGKV4-1*01	Kappa
1335	SBC210	0.7	0.2	0.2	0	IGHV5-51*01	IGKV3-15*01	Kappa
1338	SBC246	0.7	0.2	0.1	0	IGHV2-5*02	IGLV3-25*03	Lambda
1245	SBC236	2.1	0.15	0	0	IGHV3-11*01	IGLV3-10*01	Lambda
1337	SBC210	1.6	0.1	0	0	IGHV4-59*08	IGLV2-8*01	Lambda
1012	SBC257	nd	nd	nd	1.8	IGHV3-23*04	IGLV1-51*01	Lambda
1070	SBC230	0.3	0.13	0.5	0.11	IGHV3-7*01	IGKV1-6*01	Kappa
1087	SBC230	0.14	0.06	0.06	0.06	IGHV4-59*01	IGKV1D-16*01	Kappa
1218	SBC241	1.0	0.09	0.06	0.04	IGHV3-30*04	IGLV2-14*01	Lambda
1230	SBC241	0.1	0.3	0.07	0.06	IGHV3-21*01	IGLV3-25*02	Lambda
1232	SBC241	0.2	0.2	0.1	0.1	IGHV1-2*02	IGKV3-20*01	Kappa
1242	SBC246	1.9	0	0	0	IGHV5-51*01	IGKV4-1*01	Kappa
1339	SBC246	0.3	0	0	0	IGHV5-51*01	IGKV4-1*01	Kappa
1341	SBC246	0.6	0	0	0	IGHV3-73*01	IGLV3-10*01	Lambda
1347	SBC241	1.2	0	0	0	IGHV3-73*01	IGLV1-44*01	Lambda
1361	SBC236	2.0	0	0	0	IGHV3-23*04	IGKV2D-29*01	Kappa

Specificity assessment using primary transfected cell supernatants; ELISA value measured as OD450 nm; 0 = OD below BSA background; nd = not done.

**Table 2:** Affinities of selected broad and narrow spectrum mAbs.

mAb TRL#	ELISA K_D_ (nM)			
	*S*.*a*. HU beta	*A*.*b*. IHF alpha	*K*.*p*. IHF alpha	*H*.*i*. IHF alpha
1068	0.012	0.005	0.02	24.08
1330	0.064	0.060	0.06	0.08
1335	>305	No Binding	No Binding	No Binding
1337	5.5	No Binding	No Binding	No Binding
1338	26.9	No Binding	No Binding	No Binding
1341	No Binding	No Binding	No Binding	No Binding
1347	>300	>196	No Binding	No Binding
1361	6.1	>258	No Binding	No Binding

K_D_ is estimated using the point of inflection of a 4-paramter logistic curve fit to the ELISA binding curves; (*S*.*a = S*. *aureus; P*.*a*. *= P*. *aeruginosa; A*.*b*. *= A*. *baumannii; K*.*p*. *= K*. *pneumoniae; H*.*i*. *= H*. *influenzae*).

Two of the mAbs (#1330 and #1215) were cloned from the same donor in screening efforts on different dates and have nearly identical sequences; the difference of 1 amino acid (residue 4 of the VL) possibly reflects an error in RT-PCR amplification of the same mAb. By contrast, #1261 and #1262 have very different sequences despite having been cloned in the same experiment from the same donor; the same holds true for #1242 and #1339. Full sequences for all 21 mAbs are provided in the Supplementary Information.

**Fig 1:**
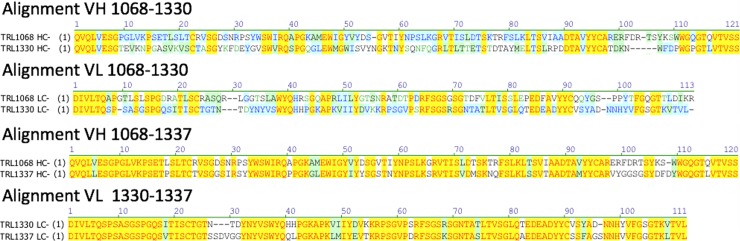
Sequence alignments. For the mAbs binding to the most conserved region of *S*. *aureus* HU, sequence alignments are shown. TRL1068 and TRL1330 are quite different but #1337 has high VH homology with TRL1068 and high VL homology with TRL1330. Yellow highlighting indicates identity over all strains; blue highlighting indicates partial identity; green highlighting indicates conservative substitution.

Germlines were determined using the VBase2 database which is an integrative database of germline variable genes from the immunoglobulin loci of human and mouse from the EMBL-Bank [23]. All of the cloned mAbs are somatically mutated from germline and include amino acids from the N-region (random additions of amino acids) between the VDJ and VJ rearrangements. TRL1068 and TRL1330 are the only mAbs with high affinity against *S*. *aureus*, *P*. *aeruginosa*, *K*. *pneumoniae*, *A baumannii and H*. *influenzae* DNABII homologs. These two mAbs use different VH and VL gene segments and consequently are very different in their amino acid sequences. Interestingly, although #1337 was cloned from another donor, this mAb uses the same VH segment as TRL1068 and the same VL segment as TRL1330. These three mAbs bind to the same region of the target as defined by peptide epitope mapping, suggesting significant constraints on the germline origins for mAbs to this region. All the other mAbs, derived from diverse germline sequences, bind to different regions. Although the antibody heavy chain by itself can achieve high affinity [24], in this case the specific heavy and light chain pairing is important since #1337 has approximately 1,000-fold weaker binding compared to TRL1068 and TRL1330. The sequence alignments for these three mAbs are shown in Fig 1; see Supplemental Information for the full sequences.

**Fig 2:**
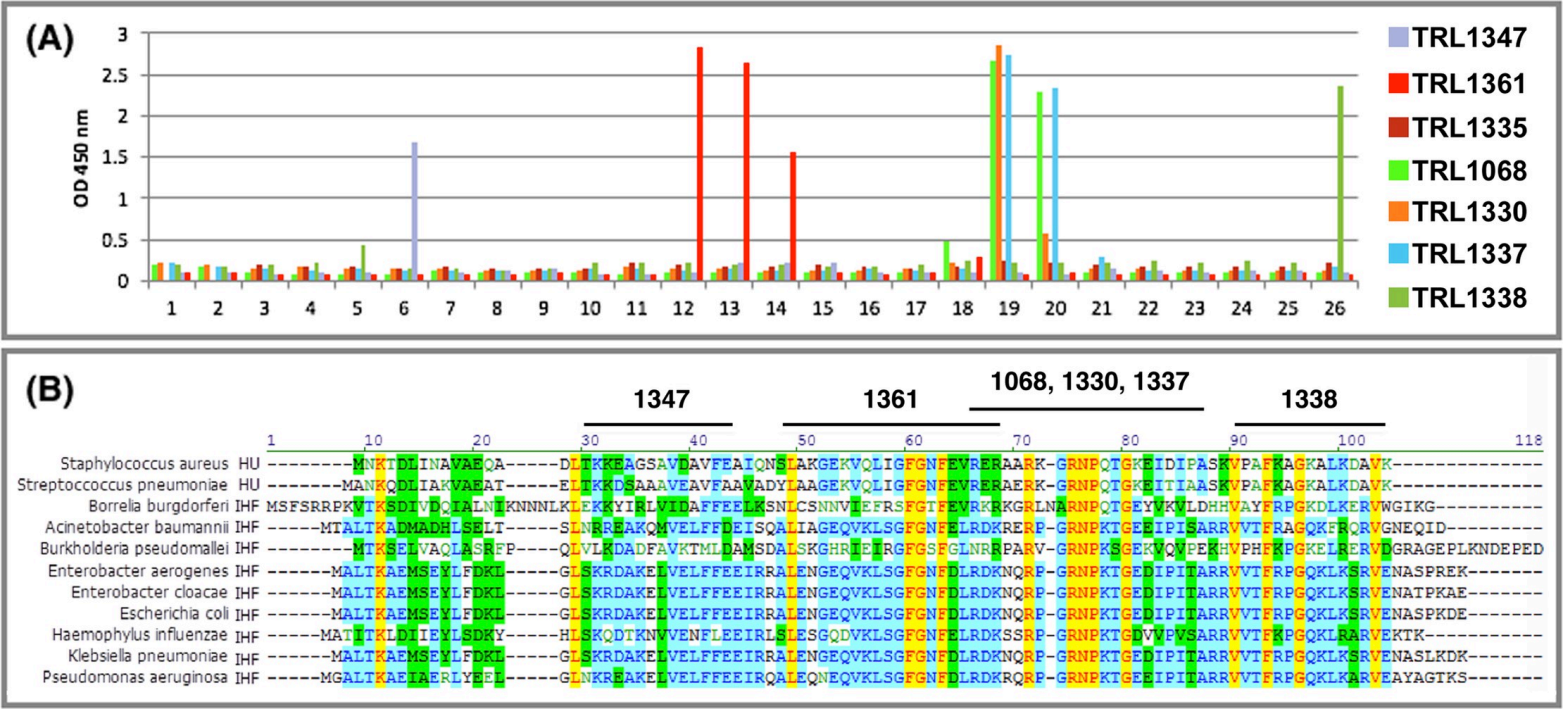
Epitope mapping. For representative mAbs, epitope mapping data are shown. (A) Binding for each mAb was quantified against a panel of 26 15-mer peptides (overlap of 3 residues) spanning the sequence of the *S*. *aureus* HU. TRL1335 is a weak binding control with no well defined epitope. (B) The lead clinical candidate and backup mAbs, TRL1068 and TRL1330, bind to the most highly conserved region. Yellow highlighting indicates identity over all strains; blue highlighting indicates partial identity; green highlighting indicates conservative substitution.

Epitope mapping was performed for seven of the mAbs against a panel of peptides as previously described for TLR1068 [19]. Each mAb was exposed to a series of 26 overlapping 15-mer peptides, offset by 3, spanning the full sequence of the HU protein from *S*. *aureus*. Binding was assessed by semiquantitative single concentration ELISA (in duplicates). Fig 2A shows the binding profiles. For most of the mAbs, two or three of the overlapping peptides were recognized, providing an internal control against non-specific binding. The epitopes thereby defined were mapped onto the HU sequence of *S*. *aureus* as shown in Fig 2B which also shows the sequence conservation across diverse gram positive and gram negative DNABII proteins.

## Discussion

Since the mammalian proteome does not have any homologs to DNABII proteins, the presence of memory B-cells producing anti-DNABII antibodies in an individual donor’s repertoire suggests that the individual has been exposed to the bacterial antigen, consistent with its presence extracellularly as part of a biofilm [25]. BLAST searching revealed presence of DNABII family proteins within both gram positive and negative bacteria; no epitope mimics from outside the family were discovered by this extensive search. Accordingly, finding such mAbs in all twenty healthy blood donors surveyed suggests that the majority of healthy individuals have been exposed to bacterial biofilm at some point in their lives. Although the probe used to identify these antibodies was the DNABII homolog from *S*. *aureus* [19], other species of bacteria may also have contributed to the immune response given the degree of sequence conservation in this family. At the time of blood sampling, the donors were healthy (no reported infection is a condition to be eligible for donating blood at the Stanford Blood Center), and the anti-DNABII antibodies in plasma were below the detection limit of our ELISA assay. The single B-cell CellSpot assay used to identify the mAbs is orders of magnitude more sensitive and reflects the full history of exposure to the antigen.

We cloned a total of 21 mAbs based on binding to at least one of the DNABII proteins used in the primary screen. The screening assay is biased in favor of mAbs with an affinity in the low nanomolar to picomolar range [22]; each of the mAbs is a somatically mutated mAb with specificity for some epitope within the DNABII family. As summarized in Table 2, the mAbs with the strongest qualitative binding (Table 1) do indeed have affinities in the low nanomolar range for at least one family member. However, only 2 unique mAbs (TRL1068 and TRL1330) exhibit broad-spectrum binding to homologs from gram positive and gram negative bacteria and low picomolar affinity to nearly all of the target proteins; #1215 has the identical variable region sequence as #1330 and was cloned from the same donor in different screening experiments on different days. Eight of the mAbs have a narrower spectrum and the remaining ten bind to only one of the four DNABII variants tested. High affinity to the target is a key feature needed for extraction of the scaffolding proteins from the biofilm matrix. Only 2 unique mAbs of the 21 meet the low picomolar affinity threshold. While it is not clear if the other mAbs are able to disrupt biofilm at a low level, their weak affinity and restricted range of specificity suggests that they are not curative. Nonetheless, we speculate that such mAbs may have arisen following exposure to biofilm reservoirs which may remain cryptic or represent an early colonization stage preceding a clinically apparent infection.

Chronic inflammation is a pervasive feature of most if not all age-related diseases, generally attributed to immune response to debris from damaged and senescent cells. Franceschi and colleagues have termed this phenomenon “inflammaging” to suggest a causal link between chronic inflammation and the various pathologies of aging [26]. We hypothesize that shedding of bacteria from cryptic biofilm reservoirs provides a plausible mechanism contributing to low-grade, chronic inflammaging. Rheumatoid arthritis (RA) is an example of particular interest in light of its high incidence. A direct link to bacterial infection has been elusive, but it is well established that periodontal disease is more frequent and more severe in patients with RA compared to controls [27]. It is noteworthy that in a clinical study of treatment to reduce periodontal disease, the mean RA disease activity score was reduced (P<0.001). The principal therapeutic target for RA treatment, TNF-alpha, was also reduced (P<0.05) in the treated patients [28]. We suggest that reducing cryptic biofilm-protected bacterial colonies will decrease the overall state of inflammation both locally and systemically and will decrease the progressive inflammation related pathology of aging.

Low grade infectious foci are difficult to eliminate. The biofilm shields the bacteria from phagocytic attack by the cellular immune system, and the sessile state renders the bacteria highly resistant to most antibiotics. Using high dose antibiotics to attack low grade infections is not justified since doing so will select for mutants that are drug resistant even in the planktonic state. Moreover, lysing bacteria while still in the biofilm contributes more of the components needed to augment the biofilm. That is, exogenous addition of DNABII proteins and eDNA has been shown to increase the average thickness of a biofilm *in vitro* [29]. Use of DNAse to disrupt biofilms is not practical since extensive degradation of the eDNA is needed to disrupt the structure. Extracting the DNABII proteins with a high affinity mAb is more practical since the targets are sparse within the matrix and even partial extraction substantially disrupts the meshwork structure as previously reported [19], [21]. TRL1068 is the first biofilm disrupting clinical candidate for which clear utility has been demonstrated in a variety of biofilm mediated infection models in rodents. Activity has been shown for both gram positive and gram negative bacteria which is important since multiple species of bacteria can cooperatively form a biofilm. Although currently in preclinical development to treat acute biofilm associated infections, broader use may be appropriate to retard the aging process.

In summary, the presence of antibodies to a key biofilm component in all twenty of the healthy blood donors we surveyed suggests that biofilms are commonly encountered, possibly as cryptic reservoirs of biofilm protected bacteria. Reducing the chronic inflammation associated with shedding of bacteria from the biofilm should be beneficial. While a longevity benefit arising from attacking such reservoirs might be difficult to measure directly, telomere shortening measured in circulating leukocytes has been linked to chronic inflammatory insults [30], and we conjecture that quantifying that parameter may provide a surrogate measure of utility for a wide range of aging-related diseases.

## Supporting information

S1 Fig Antibody sequences. Amino acid and encoding DNA sequences are provided for all of the monoclonal antibodies characterized in this paper, including both heavy and light chains fused to a human IgG1 Fc region.
(DOCX)

S1 Table Affinity calculations. ELISA binding data supporting the Kd calculation presented in Table 2 are provided here, including duplicate assays.
(XLSX)
