# Supplementary material for: Human antibody repertoire frequently includes antibodies to a bacterial biofilm associated protein
Source: PLoS One. 2019 Jul 9;14(7):e0219256. doi: 10.1371/journal.pone.0219256 (PMC6615618; doi:10.1371/journal.pone.0219256)
Supplement: S1 Fig — Amino acid and encoding DNA sequences are provided for all of the monoclonal antibodies characterized in this paper, including both heavy and light chains fused to a human IgG1 Fc region. (DOCX) [file pone.0219256.s001.docx]

**Supplemental Information 1: Amino acid and encoding DNA sequences are provided for all of the monoclonal antibodies characterized in this paper, including both heavy and light chains fused to a human IgG1 Fc region.**

**TRL1012**

**Heavy Chain Nucleotide Sequence:**

caggtgcagctggtggagtccgggggaggcttggtacagcctggggggtccctgagactttcctgtgccgcctctggattccccttcagtagttatgccatgagttgggtccgtcaggctccagggaaggggctggagtgggtctcagccatcagtggcaacggcgctgactcatattacgcagactccgtgaagggccgcttcaccacttccagagacaagtccaagaatacagtttatttgcaaatgaacagactcagggccgaggacacggccgtatattactgtgcgaaagatatgcgacggtatcattatgacagtagtggtctgcacttctggggccagggaaccctggtcaccgtctcctca

**Heavy Chain Amino Acid Sequence:**

QVQLVESGGGLVQPGGSLRLSCAASGFPFSSYAMSWVRQAPGKGLEWVSAISGNGADSYYADSVKGRFTTSRDKSKNTVYLQMNRLRAEDTAVYYCAKDMRRYHYDSSGLHFWGQGTLVTVSS

**Heavy Chain Nucleotide and Amino Acid Sequences:**

caggtgcagctggtggagtccgggggaggcttggtacagcctggggggtccctgagactt

Q V Q L V E S G G G L V Q P G G S L R L

tcctgtgccgcctctggattccccttcagtagttatgccatgagttgggtccgtcaggct

S C A A S G F P F S S Y A M S W V R Q A

ccagggaaggggctggagtgggtctcagccatcagtggcaacggcgctgactcatattac

P G K G L E W V S A I S G N G A D S Y Y

gcagactccgtgaagggccgcttcaccacttccagagacaagtccaagaatacagtttat

A D S V K G R F T T S R D K S K N T V Y

ttgcaaatgaacagactcagggccgaggacacggccgtatattactgtgcgaaagatatg

L Q M N R L R A E D T A V Y Y C A K D M

cgacggtatcattatgacagtagtggtctgcacttctggggccagggaaccctggtcacc

R R Y H Y D S S G L H F W G Q G T L V T

gtctcctca

V S S

**Light Chain Nucleotide Sequence:**

gatatcatgctgactcagcccccctcagtgtctgcggcccccggacagaaggtcaccatctcctgctctggaagcagctccaacattgggacgaattatgtgtcctggttccagcaggtcccaggaacagcccccaaattcctcatttatgacaattataaacgaccctcagaaactcctgaccgattctctggctccaagtctggcacgtcggccaccctggacatcaccggactccagactggggacgaggccaattattactgcgcaacatgggacagtagcctgagtgcttgggtgttcggcggagggaccaaggtgaccgtcctg

**Light Chain Amino Acid Sequence:**

DIMLTQPPSVSAAPGQKVTISCSGSSSNIGTNYVSWFQQVPGTAPKFLIYDNYKRPSETPDRFSGSKSGTSATLDITGLQTGDEANYYCATWDSSLSA WVFGGGTKVTVL

**Light Chain Nucleotide and Amino Acid Sequences:**

gatatcatgctgactcagcccccctcagtgtctgcggcccccggacagaaggtcaccatc

D I M L T Q P P S V S A A P G Q K V T I

tcctgctctggaagcagctccaacattgggacgaattatgtgtcctggttccagcaggtc

S C S G S S S N I G T N Y V S W F Q Q V

ccaggaacagcccccaaattcctcatttatgacaattataaacgaccctcagaaactcct

P G T A P K F L I Y D N Y K R P S E T P

gaccgattctctggctccaagtctggcacgtcggccaccctggacatcaccggactccag

D R F S G S K S G T S A T L D I T G L Q

actggggacgaggccaattattactgcgcaacatgggacagtagcctgagtgcttgggtg

T G D E A N Y Y C A T W D S S L S A W V

ttcggcggagggaccaaggtgaccgtcctg

F G G G T K V T V L

**TRL1068**

**Heavy Chain Nucleotide Sequence:**

caggtgcagctggtggagtccggcccaggactggtgaagccttcggagaccctgtccctcacctgcagggtctctggtgactccaatcggccttcctactggagctggatcaggcaggccccagggaaggcaatggagtggataggttatgtctatgacagtggggtcaccatctacaatccctccctcaagggtcgagtcacaatatcactagacacgtcgaagacgcggttctccctgaaactgacctctgtgatcgctgcggacacggccgtatattattgtgcgcgagaacgttttgatcggacatcgtataagagttggtggggccagggaacgcaggtcaccgtctcctca

**Heavy Chain Amino Acid Sequence:**

QVQLVESGPGLVKPSETLSLTCRVSGDSNRPSYWSWIRQAPGKAMEWIGYVYDSGVTIYNPSLKGRVTISLDTSKTRFSLKLTSVIAADTAVYYCARERFDRTSYKSWWGQGTQVTVSS

**Heavy Chain Nucleotide and Amino Acid Sequences:**

caggtgcagctggtggagtccggcccaggactggtgaagccttcggagaccctgtccctc

Q V Q L V E S G P G L V K P S E T L S L

acctgcagggtctctggtgactccaatcggccttcctactggagctggatcaggcaggcc

T C R V S G D S N R P S Y W S W I R Q A

ccagggaaggcaatggagtggataggttatgtctatgacagtggggtcaccatctacaat

P G K A M E W I G Y V Y D S G V T I Y N

ccctccctcaagggtcgagtcacaatatcactagacacgtcgaagacgcggttctccctg

P S L K G R V T I S L D T S K T R F S L

aaactgacctctgtgatcgctgcggacacggccgtatattattgtgcgcgagaacgtttt

K L T S V I A A D T A V Y Y C A R E R F

gatcggacatcgtataagagttggtggggccagggaacgcaggtcaccgtctcctca

D R T S Y K S W W G Q G T Q V T V S S

**Light Chain Nucleotide Sequence:**

gatatcgtgctgactcaggccccaggcactctgtctttgtctccaggggacagagccaccctctcctgtagggccagtcagcgtcttggcggcacgtccttagcctggtaccagcacagatctggccaggctcccaggctcatcctctacggaacttcaaacagggccactgacacccctgacaggtttagtggcagtgggtctgggacagacttcgttctcaccatcagttccctggagcctgaagattttgcagtgtattactgtcagcaatatggcagcccaccgtacacttttggccaggggaccactctggacatcaaa

**Light Chain Amino Acid Sequence:**

DIVLTQAPGTLSLSPGDRATLSCRASQRLGGTSLAWYQHRSGQAPRLILYGTSNRATDTPDRFSGSGSGTDFVLTISSLEPEDFAVYYCQQYGSPPYTFGQGTTLDIK

**Light Chain Nucleotide and Amino Acid Sequences:**

gatatcgtgctgactcaggccccaggcactctgtctttgtctccaggggacagagccacc

D I V L T Q A P G T L S L S P G D R A T

ctctcctgtagggccagtcagcgtcttggcggcacgtccttagcctggtaccagcacaga

L S C R A S Q R L G G T S L A W Y Q H R

tctggccaggctcccaggctcatcctctacggaacttcaaacagggccactgacacccct

S G Q A P R L I L Y G T S N R A T D T P

gacaggtttagtggcagtgggtctgggacagacttcgttctcaccatcagttccctggag

D R F S G S G S G T D F V L T I S S L E

cctgaagattttgcagtgtattactgtcagcaatatggcagcccaccgtacacttttggc

P E D F A V Y Y C Q Q Y G S P P Y T F G

caggggaccactctggacatcaaa

Q G T T L D I K

**TRL1070**

**Heavy Chain Nucleotide Sequence:**

caggtgcagctggtgcagtctgggggaaccttggtccagccgggggggtccctgagactctcctgtgcagcctctggattcacctttagttactactcgatgagctgggtccgccaggctccagggaaggggctggagtgggtggccaacataaagcacgatggaactgagagaaattatgtggactctgtgaagggccgattcaccatctccagagacaacagcgagaagtctctttacctgcaaatgaacagcctgagagccgaggacacggctgtgtattactgtgcgaagtattattatggtgccgggactaattatccccttaagtactggggccagggaacccgggtcaccgtctcctca

**Heavy Chain Amino Acid Sequence:**

QVQLVQSGGTLVQPGGSLRLSCAASGFTFSYYSMSWVRQAPGKGLEWVANIKHDGTERNYVDSVKGRFTISRDNSEKSLYLQMNSLRAEDTAVYYCAKYYYGAGTNYPLKYWGQGTRVTVSS

**Heavy Chain Nucleotide and Amino Acid Sequences:**

caggtgcagctggtgcagtctgggggaaccttggtccagccgggggggtccctgagactc

Q V Q L V Q S G G T L V Q P G G S L R L

tcctgtgcagcctctggattcacctttagttactactcgatgagctgggtccgccaggct

S C A A S G F T F S Y Y S M S W V R Q A

ccagggaaggggctggagtgggtggccaacataaagcacgatggaactgagagaaattat

P G K G L E W V A N I K H D G T E R N Y

gtggactctgtgaagggccgattcaccatctccagagacaacagcgagaagtctctttac

V D S V K G R F T I S R D N S E K S L Y

ctgcaaatgaacagcctgagagccgaggacacggctgtgtattactgtgcgaagtattat

L Q M N S L R A E D T A V Y Y C A K Y Y

tatggtgccgggactaattatccccttaagtactggggccagggaacccgggtcaccgtc

Y G A G T N Y P L K Y W G Q G T R V T V

tcctca

S S

**Light Chain Nucleotide Sequence:**

gatatcctgatgacccagtctccatcctccctgtctgcatctgtaggagacagagtcaccatcacttgccgggcaagtcagggcattagaaatgatttaggctggtatcagcagaaaccagggaaagcccctaagctcctgatctatgctgcatccagtttacaaagtggggtcccatcaaggttcagcggcagtggatctggcacagatttcactctcaccatcagcagcctgcagcctgaagattttgcaacttattactgtctacaagattacaattacccgctcactttcggcggagggaccaaggtggagatcaaacga

**Light Chain Amino Acid Sequence:**

DILMTQSPSSLSASVGDRVTITCRASQGIRNDLGWYQQKPGKAPKLLIYAASSLQSGVPSRFSGSGSGTDFTLTISSLQPEDFATYYCLQDYNYPLTFGGGTKVEIKR

**Light Chain Nucleotide and Amino Acid Sequences:**

gatatcctgatgacccagtctccatcctccctgtctgcatctgtaggagacagagtcacc

D I L M T Q S P S S L S A S V G D R V T

atcacttgccgggcaagtcagggcattagaaatgatttaggctggtatcagcagaaacca

I T C R A S Q G I R N D L G W Y Q Q K P

gggaaagcccctaagctcctgatctatgctgcatccagtttacaaagtggggtcccatca

G K A P K L L I Y A A S S L Q S G V P S

aggttcagcggcagtggatctggcacagatttcactctcaccatcagcagcctgcagcct

R F S G S G S G T D F T L T I S S L Q P

gaagattttgcaacttattactgtctacaagattacaattacccgctcactttcggcgga

E D F A T Y Y C L Q D Y N Y P L T F G G

gggaccaaggtggagatcaaacga

G T K V E I K R

**TRL1087**

**Heavy Chain Nucleotide Sequence:**

caggtgcagctgctcgagtcaggcccaggcctggttaggccctcggacaccctgtccctcacctgcactttttccgctgacctcagcaccaacgcctattggacctggatccggcagcccccaggaaagggactggagtggattggctatatgtctcatagtgggggaagggattacaatccctccttcaaccggcgagtcaccatttcagtggacacgtcgaagaaccaggttttcttgaggctgacgtcagtgacctctgcggacacggccgtctatttctgtgtgagagaagtcggcagttactacgactactggggccagggaatcctggtcaccgtctcctca

**Heavy Chain Amino Acid Sequence:**

QVQLLESGPGLVRPSDTLSLTCTFSADLSTNAYWTWIRQPPGKGLEWIGYMSHSGGRDYNPSFNRRVTISVDTSKNQVFLRLTSVTSADTAVYFCVREVGSYYDYWGQGILVTVSS

**Heavy Chain Nucleotide and Amino Acid Sequences:**

caggtgcagctgctcgagtcaggcccaggcctggttaggccctcggacaccctgtccctc

Q V Q L L E S G P G L V R P S D T L S L

acctgcactttttccgctgacctcagcaccaacgcctattggacctggatccggcagccc

T C T F S A D L S T N A Y W T W I R Q P

ccaggaaagggactggagtggattggctatatgtctcatagtgggggaagggattacaat

P G K G L E W I G Y M S H S G G R D Y N

ccctccttcaaccggcgagtcaccatttcagtggacacgtcgaagaaccaggttttcttg

P S F N R R V T I S V D T S K N Q V F L

aggctgacgtcagtgacctctgcggacacggccgtctatttctgtgtgagagaagtcggc

R L T S V T S A D T A V Y F C V R E V G

agttactacgactactggggccagggaatcctggtcaccgtctcctca

S Y Y D Y W G Q G I L V T V S S

**Light Chain Nucleotide Sequence:**

gatatcgagatgacccagtctccatcctctttgtctgcatctgtcggagacagaatcaccatcacttgtcgggcgagtcagggtattagcacctggttagcctggtatcagcagaaaccggggaaagcccctaagtccctgatcttttctacgtccagcctgcatagtggggtcccctcaaagttcagcggcagtgggtctgggacagacttcactctcaccatcaccaacctgcagcctgaagattttgcaacttattactgccaacagaaatgggagaccccttatagttttggccaggggaccaagctggacatgatacga

**Light Chain Amino Acid Sequence:**

DIEMTQSPSSLSASVGDRITITCRASQGISTWLAWYQQKPGKAPKSLIFSTSSLHSGVPSKFSGSGSGTDFTLTITNLQPEDFATYYCQQKWETPYSFGQGTKLDMIR

**Light Chain Nucleotide and Amino Acid Sequences:**

gatatcgagatgacccagtctccatcctctttgtctgcatctgtcggagacagaatcacc

D I E M T Q S P S S L S A S V G D R I T

atcacttgtcgggcgagtcagggtattagcacctggttagcctggtatcagcagaaaccg

I T C R A S Q G I S T W L A W Y Q Q K P

gggaaagcccctaagtccctgatcttttctacgtccagcctgcatagtggggtcccctca

G K A P K S L I F S T S S L H S G V P S

aagttcagcggcagtgggtctgggacagacttcactctcaccatcaccaacctgcagcct

K F S G S G S G T D F T L T I T N L Q P

gaagattttgcaacttattactgccaacagaaatgggagaccccttatagttttggccag

E D F A T Y Y C Q Q K W E T P Y S F G Q

gggaccaagctggacatgatacga

G T K L D M I R

**TRL1215**

**Heavy Chain Nucleotide Sequence:**

caggtgcagctggtggagtctggaactgaggtgaagaaccctggagcctcagtgaaggtctcctgcacggcctctggttacaaatttgacgaatatggtgtcagttgggtgcgacagtcccctggacaaggacttgagtggatgggatggatcagtgtttataatggcaagacaaactatagccagaactttcagggcagactcaccctgaccacagagacatccaccgacacagcctacatggagcttacgagcctcagacctgacgacacggccgtctattactgtgcgacagacaaaaactggttcgacccctggggcccgggaaccctggtcaccgtctcctca

**Heavy Chain Amino Acid Sequence:**

QVQLVESGTEVKNPGASVKVSCTASGYKFDEYGVSWVRQSPGQGLEWMGWISVYNGKTNYSQNFQGRLTLTTETSTDTAYMELTSLRPDDTAVYYCATDKNWFDPWGPGTLVTVSS

**Heavy Chain Nucleotide and Amino Acid Sequences:**

caggtgcagctggtggagtctggaactgaggtgaagaaccctggagcctcagtgaaggtc

Q V Q L V E S G T E V K N P G A S V K V

tcctgcacggcctctggttacaaatttgacgaatatggtgtcagttgggtgcgacagtcc

S C T A S G Y K F D E Y G V S W V R Q S

cctggacaaggacttgagtggatgggatggatcagtgtttataatggcaagacaaactat

P G Q G L E W M G W I S V Y N G K T N Y

agccagaactttcagggcagactcaccctgaccacagagacatccaccgacacagcctac

S Q N F Q G R L T L T T E T S T D T A Y

atggagcttacgagcctcagacctgacgacacggccgtctattactgtgcgacagacaaa

M E L T S L R P D D T A V Y Y C A T D K

aactggttcgacccctggggcccgggaaccctggtcaccgtctcctca

N W F D P W G P G T L V T V S S

**Light Chain Nucleotide Sequence:**

gatatcgtgatgacccagtctccctccgcgtccgggtctcctggacagtcaatcaccatctcctgcactggaaccaacactgattataattatgtttcctggtaccagcaccaccccggcaaagcccccaaagtcattatttatgacgtcaaaaagcggccctcgggggtccctagtcgcttctctggctccaggtctggcaacacggccaccctgaccgtctctgggctccagactgaggatgaggctgattattattgtgtctcatatgcagacaacaatcattatgtcttcggaagtgggaccaaggtcaccgtcctg

**Light Chain Amino Acid Sequence:**

DIVMTQSPSASGSPGQSITISCTGTNTDYNYVSWYQHHPGKAPKVIIYDVKKRPSGVPSRFSGSRSGNTATLTVSGLQTEDEADYYCVSYADNNHYVFGSGTKVTVL

**Light Chain Nucleotide and Amino Acid Sequences:**

gatatcgtgatgacccagtctccctccgcgtccgggtctcctggacagtcaatcaccatc

D I V M T Q S P S A S G S P G Q S I T I

tcctgcactggaaccaacactgattataattatgtttcctggtaccagcaccaccccggc

S C T G T N T D Y N Y V S W Y Q H H P G

aaagcccccaaagtcattatttatgacgtcaaaaagcggccctcgggggtccctagtcgc

K A P K V I I Y D V K K R P S G V P S R

ttctctggctccaggtctggcaacacggccaccctgaccgtctctgggctccagactgag

F S G S R S G N T A T L T V S G L Q T E

gatgaggctgattattattgtgtctcatatgcagacaacaatcattatgtcttcggaagt

D E A D Y Y C V S Y A D N N H Y V F G S

gggaccaaggtcaccgtcctg

G T K V T V L

**TRL1216**

**Heavy Chain Nucleotide Sequence:**

caggtgcagctggtggagtccgggggaggcgtggtccagcctggagggtccctgagagtctcctgtgcagcctctgcgttcagtttcagggattatggcatacactgggtccgccaggctccaggcaaggggctgcaatgggtggcggttatttcacatgatggaggtaagaaattctatgcagactccgtgaggggccgattcaccatctccagagacaattccgagaacacactgtatctccaaatgaacagcctgagatctgacgacacggctgtctattactgtgcgaggctcgttgccagttgcagtggttccacctgcacaacgcaacctgctgcctttgacatttggggcccagggacattggtcaccgtctcttca

**Heavy Chain Amino Acid Sequence:**

QVQLLESGADMVQPGRSLRLSCAASGFNFRTYAMHWVRQAPGKGLEWVAVMSHDGYTKYYSDSVRGQFTISRDNSKNTLYLQMNNLRPDDTAIYYCARGLTGLSVGFDYWGQGTLVTVSS

**Heavy Chain Nucleotide and Amino Acid Sequences:**

caggtgcagctggtggagtccgggggaggcgtggtccagcctggagggtccctgagagtc

Q V Q L V E S G G G V V Q P G G S L R V

tcctgtgcagcctctgcgttcagtttcagggattatggcatacactgggtccgccaggct

S C A A S A F S F R D Y G I H W V R Q A

ccaggcaaggggctgcaatgggtggcggttatttcacatgatggaggtaagaaattctat

P G K G L Q W V A V I S H D G G K K F Y

gcagactccgtgaggggccgattcaccatctccagagacaattccgagaacacactgtat

A D S V R G R F T I S R D N S E N T L Y

ctccaaatgaacagcctgagatctgacgacacggctgtctattactgtgcgaggctcgtt

L Q M N S L R S D D T A V Y Y C A R L V

gccagttgcagtggttccacctgcacaacgcaacctgctgcctttgacatttggggccca

A S C S G S T C T T Q P A A F D I W G P

gggacattggtcaccgtctcttca

G T L V T V S S

**Light Chain Nucleotide Sequence:**

gatatcatgctgactcagccgccctcggtgtcagtgtccccaggacaaacggccaggatcacctgctctggagatgcattgccaaaaaaatatacttattggtatcagcagaagtcaggccaggcccctgttctgctcatctatgaggacaggaaacgaccctccgagatccctgagagattctctgccttcacctcatggacgacggccaccttgactatcactggggcccaggtgagagatgaagctgactactactgttattcaacagacatcagtggtgatataggagtgttcggcggagggaccaagctgaccgtccta

**Light Chain Amino Acid Sequence:**

DIVLTQSASVSGSPGQSITISCTGTSSDVGGYNYVSWYQQHPGKAPKLMIYDVTTRPSGVSDRFSGSKSGNTASLTISGLQAEDEADYYCSSYSSGSTPALFGGGTQLTVL

**Light Chain Nucleotide and Amino Acid Sequences:**

gatatcatgctgactcagccgccctcggtgtcagtgtccccaggacaaacggccaggatc

D I M L T Q P P S V S V S P G Q T A R I

acctgctctggagatgcattgccaaaaaaatatacttattggtatcagcagaagtcaggc

T C S G D A L P K K Y T Y W Y Q Q K S G

caggcccctgttctgctcatctatgaggacaggaaacgaccctccgagatccctgagaga

Q A P V L L I Y E D R K R P S E I P E R

ttctctgccttcacctcatggacgacggccaccttgactatcactggggcccaggtgaga

F S A F T S W T T A T L T I T G A Q V R

gatgaagctgactactactgttattcaacagacatcagtggtgatataggagtgttcggc

D E A D Y Y C Y S T D I S G D I G V F G

ggagggaccaagctgaccgtccta

G G T K L T V L

**TRL1218**

**Heavy Chain Nucleotide Sequence:**

cagctgcagctggtggagtcaggggcagacatggtccagcctgggaggtccctgagactctcctgtgcagcctctggattcaacttcaggacctatgctatgcactgggtccgccaggctccaggcaaggggctggagtgggtggcagttatgtcacatgatggatacactaaatactactcagactccgtgaggggccaattcaccatctccagagacaattccaagaacacactgtatctgcaaatgaacaacctgagacctgacgacacggctatatattactgtgcgagaggcctttcaggtctgtcagtcggttttgactactggggccagggaaccctggtcaccgtctcctca

**Heavy Chain Amino Acid Sequence:**

QLQLVESGADMVQPGRSLRLSCAASGFNFRTYAMHWVRQAPGKGLEWVAVMSHDGYTKYYSDSVRGQFTISRDNSKNTLYLQMNNLRPDDTAIYYCARGLSGLSVGFDYWGQGTLVTVSS

**Heavy Chain Nucleotide and Amino Acid Sequences:**

cagctgcagctggtggagtcaggggcagacatggtccagcctgggaggtccctgagactc

Q L Q L V E S G A D M V Q P G R S L R L

tcctgtgcagcctctggattcaacttcaggacctatgctatgcactgggtccgccaggct

S C A A S G F N F R T Y A M H W V R Q A

ccaggcaaggggctggagtgggtggcagttatgtcacatgatggatacactaaatactac

P G K G L E W V A V M S H D G Y T K Y Y

tcagactccgtgaggggccaattcaccatctccagagacaattccaagaacacactgtat

S D S V R G Q F T I S R D N S K N T L Y

ctgcaaatgaacaacctgagacctgacgacacggctatatattactgtgcgagaggcctt

L Q M N N L R P D D T A I Y Y C A R G L

tcaggtctgtcagtcggttttgactactggggccagggaaccctggtcaccgtctcctca

S G L S V G F D Y W G Q G T L V T V S S

**Light Chain Nucleotide Sequence:**

gatatcgtgctgactcagtcggcctccgtgtctgggtctcctggacagtcgatcaccatctcctgcactggaaccagcagtgacgttggtggatataactatgtctcctggtaccaacaacacccaggcaaagcccccaaactcatgatttatgatgtcactactcggccttcaggggtttctgatcgcttctctggctccaagtctggcaacacggcctccctgaccatctctgggctgcaggctgaggacgaggctgattattattgcagctcatattcaagcggctccacacctgctctgtttggggggggcacccagctgaccgtcctc

**Light Chain Amino Acid Sequence:**

DIVVDQSPSVSGSPGQSITISCTGTSSDVGGYNYVSWYQQHPGKAPKLMIYDVTTRPSGVSDRFSGSKSGNTASLTISGLQAEDEADYYCSSYSSGSTPALFGGGTQLTVL

**Light Chain Nucleotide and Amino Acid Sequences:**

gatattgtggtggatcaatctccctccgtgtctgggtctcctggacagtcgatcaccatc

D I V V D Q S P S V S G S P G Q S I T I

tcctgcactggaaccagcagtgacgttggtggatataactatgtctcctggtaccaacaa

S C T G T S S D V G G Y N Y V S W Y Q Q

cacccaggcaaagcccccaaactcatgatttatgatgtcactactcggccttcaggggtt

H P G K A P K L M I Y D V T T R P S G V

tctgatcgcttctctggctccaagtctggcaacacggcctccctgaccatctctgggctg

S D R F S G S K S G N T A S L T I S G L

caggctgaggacgaggctgattattattgcagctcatattcaagcggctccacacctgct

Q A E D E A D Y Y C S S Y S S G S T P A

ctgtttggggggggcacccagctgaccgtcctc

L F G G G T Q L T V L

**TRL1230**

**Heavy Chain Nucleotide Sequence:**

caggtgcagctggtgcagtctgggggaggcctggtcaagcctggggggtccctgagactctcctgtggagcctctggatttaacctcagtagttatagcatgaactgggtccgccaggctccagggaaggggctggagtgggtctcatccattagtagtagaagtagttacatatactatgcagactcagtgcagggccgattcaccatctccagagacaacgccaagaactcactgtatctgcaaatgaacagcctgagagccgaggacacggctatatattactgtgcgagagtatctccgtccacctattattattatggtatggacgtctggggccaag

ggaccacggtcaccgtctcctca

**Heavy Chain Amino Acid Sequence:**

QVQLVQSGGGLVKPGGSLRLSCGASGFNLSSYSMNWVRQAPGKGLEWVSSISSRSSYIYYADSVQGRFTISRDNAKNSLYLQMNSLRAEDTAIYYCARVSPSTYYYYGMDVWGQGTTVTVSS

**Heavy Chain Nucleotide and Amino Acid Sequences:**

caggtgcagctggtgcagtctgggggaggcctggtcaagcctggggggtccctgagactc

Q V Q L V Q S G G G L V K P G G S L R L

tcctgtggagcctctggatttaacctcagtagttatagcatgaactgggtccgccaggct

S C G A S G F N L S S Y S M N W V R Q A

ccagggaaggggctggagtgggtctcatccattagtagtagaagtagttacatatactat

P G K G L E W V S S I S S R S S Y I Y Y

gcagactcagtgcagggccgattcaccatctccagagacaacgccaagaactcactgtat

A D S V Q G R F T I S R D N A K N S L Y

ctgcaaatgaacagcctgagagccgaggacacggctatatattactgtgcgagagtatct

L Q M N S L R A E D T A I Y Y C A R V S

ccgtccacctattattattatggtatggacgtctggggccaagggaccacggtcaccgtc

P S T Y Y Y Y G M D V W G Q G T T V T V

tcctca

S S

**Light Chain Nucleotide Sequence:**

gatatcgtactcactcagccgtcctcggtgtcagtgtccccaggacagacggccaggatcacctgctctggagatgaattgccaaagcaatatgcttattggtaccagcagaagccaggccaggcccctgtgttggtaatatataaagacaatgagaggccctcagggatccctgagcgattctctggctccagctcagggacaacagtcacgttgaccatcagtggagtccaggcagaagacgaggctgactattactgtcaatcagcagacagtagtggtacttatgtggtgttcggcggagggaccaagctgaccgtccta

**Light Chain Amino Acid Sequence:**

DIVLTQPSSVSVSPGQTARITCSGDELPKQYAYWYQQKPGQAPVLVIYKDNERPSGIPERFSGSSSGTTVTLTISGVQAEDEADYYCQSADSSGTYVVFGGGTKLTVL

**Light Chain Nucleotide and Amino Acid Sequences:**

gatatcgtactcactcagccgtcctcggtgtcagtgtccccaggacagacggccaggatc

D I V L T Q P S S V S V S P G Q T A R I

acctgctctggagatgaattgccaaagcaatatgcttattggtaccagcagaagccaggc

T C S G D E L P K Q Y A Y W Y Q Q K P G

caggcccctgtgttggtaatatataaagacaatgagaggccctcagggatccctgagcga

Q A P V L V I Y K D N E R P S G I P E R

ttctctggctccagctcagggacaacagtcacgttgaccatcagtggagtccaggcagaa

F S G S S S G T T V T L T I S G V Q A E

gacgaggctgactattactgtcaatcagcagacagtagtggtacttatgtggtgttcggc

D E A D Y Y C Q S A D S S G T Y V V F G

ggagggaccaagctgaccgtccta

G G T K L T V L

**TRL1232**

**Heavy Chain Nucleotide Sequence:**

caggtgcagctggtggagtctggggctgaggtgaagaagcctggggccttagtgaaggtctcctgcaaggcttctggatacaccttcagcggctactatatgcactgggtgcgacaggcccctggacaagggcttgagtggatgggatggatcaaccctaagagtggtggcacaaagtatgcacagaagtttcagggccgggtcaccatgaccagggacacgtccatcagcacagcctacatggagttgagcaggctaagatctgacgacacggccgtgtatttctgtgcgagaggcggaccttcaaatttggaacgatttttggagaggttacaaccccgctacagttacgacgacaagtatgctatggacgtctggggccaagggaccacggtcaccgtctcctca

**Heavy Chain Amino Acid Sequence:**

QVQLVESGAEVKKPGALVKVSCKASGYTFSGYYMHWVRQAPGQGLEWMGWINPKSGGTKYAQKFQGRVTMTRDTSISTAYMELSRLRSDDTAVYFCARGGPSNLERFLERLQPRYSYDDKYAMDVWGQGTTVTVSS

**Heavy Chain Nucleotide and Amino Acid Sequences:**

caggtgcagctggtggagtctggggctgaggtgaagaagcctggggccttagtgaaggtc

Q V Q L V E S G A E V K K P G A L V K V

tcctgcaaggcttctggatacaccttcagcggctactatatgcactgggtgcgacaggcc

S C K A S G Y T F S G Y Y M H W V R Q A

cctggacaagggcttgagtggatgggatggatcaaccctaagagtggtggcacaaagtat

P G Q G L E W M G W I N P K S G G T K Y

gcacagaagtttcagggccgggtcaccatgaccagggacacgtccatcagcacagcctac

A Q K F Q G R V T M T R D T S I S T A Y

atggagttgagcaggctaagatctgacgacacggccgtgtatttctgtgcgagaggcgga

M E L S R L R S D D T A V Y F C A R G G

ccttcaaatttggaacgatttttggagaggttacaaccccgctacagttacgacgacaag

P S N L E R F L E R L Q P R Y S Y D D K

tatgctatggacgtctggggccaagggaccacggtcaccgtctcctca

Y A M D V W G Q G T T V T V S S

**Light Chain Nucleotide Sequence:**

gatatcgtgatgacccagtctccaggcaccctgtctttgtctccaggggcaagagccaccctctcctgcagggccagtcagagtgttagcagcatctatttagcctggtaccagcagaaacctggccaggctcccaggctcctcatctttggtgcatccagcagggccactggcatcccagacaggttcagtggcagtgggtctgggacagacttcactctcaccatcagcagactggagcctgaagattttgcagtgtattactgtcagcagtatggtagctcaccgtacacttttggccaggggaccaagctggagatcaaacgaa

**Light Chain Amino Acid Sequence:**

DIVMTQSPGTLSLSPGARATLSCRASQSVSSIYLAWYQQKPGQAPRLLIFGASSRATGIPDRFSGSGSGTDFTLTISRLEPEDFAVYYCQQYGSSPYTFGQGTKLEIKR

**Light Chain Nucleotide and Amino Acid Sequences:**

gatatcgtgatgacccagtctccaggcaccctgtctttgtctccaggggcaagagccacc

D I V M T Q S P G T L S L S P G A R A T

ctctcctgcagggccagtcagagtgttagcagcatctatttagcctggtaccagcagaaa

L S C R A S Q S V S S I Y L A W Y Q Q K

cctggccaggctcccaggctcctcatctttggtgcatccagcagggccactggcatccca

P G Q A P R L L I F G A S S R A T G I P

gacaggttcagtggcagtgggtctgggacagacttcactctcaccatcagcagactggag

D R F S G S G S G T D F T L T I S R L E

cctgaagattttgcagtgtattactgtcagcagtatggtagctcaccgtacacttttggc

P E D F A V Y Y C Q Q Y G S S P Y T F G

caggggaccaagctggagatcaaacgaa

Q G T K L E I K R

**TRL1242**

**Heavy Chain Nucleotide Sequence:**

caggtgcagctggtgcagtctggaacagaagtgaaaaagcccggggagtctctgaagatctcctgtgagggttctcgatacaactttgccaggtactggatcggctgggtgcgccagatgcccggaaaaggcctggactggatggggatcatctatcctggtgactccgataccagatacagcccgtccttccaaggccaggtcagcatctcagccgacaagtccatcagtaccgcctacctgcagtggaacagcctgaaggcctcggacaccgccatgtattattgtgcgagacttgggagcgagcttggagtggtctctgattattactttgactcctggggccagggaaccctggtcaccgtctcctca

**Heavy Chain Amino Acid Sequence:**

QVQLVQSGTEVKKPGESLKISCEGSRYNFARYWIGWVRQMPGKGLDWMGIIYPGDSDTRYSPSFQGQVSISADKSISTALQWNSLKASDTAMYYCARLGSELGVVSDYYFDSWGQGTLVTVSS

**Heavy Chain Nucleotide and Amino Acid Sequences:**

caggtgcagctggtgcagtctggaacagaagtgaaaaagcccggggagtctctgaagatc

Q V Q L V Q S G T E V K K P G E S L K I

tcctgtgagggttctcgatacaactttgccaggtactggatcggctgggtgcgccagatg

S C E G S R Y N F A R Y W I G W V R Q M

cccggaaaaggcctggactggatggggatcatctatcctggtgactccgataccagatac

P G K G L D W M G I I Y P G D S D T R Y

agcccgtccttccaaggccaggtcagcatctcagccgacaagtccatcagtaccgcctac

S P S F Q G Q V S I S A D K S I S T A Y

ctgcagtggaacagcctgaaggcctcggacaccgccatgtattattgtgcgagacttggg

L Q W N S L K A S D T A M Y Y C A R L G

agcgagcttggagtggtctctgattattactttgactcctggggccagggaaccctggtc

S E L G V V S D Y Y F D S W G Q G T L V

accgtctcctca

T V S S

**Light Chain Nucleotide Sequence:**

gatatcgtgttgactcagtctccagactccctggctgtgtctctgggcgagagggccaccatcaactgcaagtccagccagagtgttttagacaggtccaacaataagaactgtgtagcttggtaccagcagaaaccgggacagcctcctaaactgctcatttaccgggctgctacccgggaatccggggtccctgatcgattcagtggcagcgggtctgggacagacttcagtctcaccatcagcagcctgcaggctgaagatgtggcagtttatttctgtcagcaatattatagtattccgaacacttttggccaggggaccaagctggagatcaaacga

**Light Chain Amino Acid Sequence:**

DIVLTQSPDSLAVSLGERATINCKSSQSVLDRSNNKNCVAWYQQKPGQPPKLLIYRAATRESGVPDRFSGSGSGTDFSLTISSLQAEDVAVYFCQQYYSIPNTFGQGTKLEIKR

**Light Chain Nucleotide and Amino Acid Sequences:**

gatatcgtgttgactcagtctccagactccctggctgtgtctctgggcgagagggccacc

D I V L T Q S P D S L A V S L G E R A T

atcaactgcaagtccagccagagtgttttagacaggtccaacaataagaactgtgtagct

I N C K S S Q S V L D R S N N K N C V A

tggtaccagcagaaaccgggacagcctcctaaactgctcatttaccgggctgctacccgg

W Y Q Q K P G Q P P K L L I Y R A A T R

gaatccggggtccctgatcgattcagtggcagcgggtctgggacagacttcagtctcacc

E S G V P D R F S G S G S G T D F S L T

atcagcagcctgcaggctgaagatgtggcagtttatttctgtcagcaatattatagtatt

I S S L Q A E D V A V Y F C Q Q Y Y S I

ccgaacacttttggccaggggaccaagctggagatcaaacga

P N T F G Q G T K L E I K R

**TRL1245**

**Heavy Chain Nucleotide Sequence:**

caggtgcagctggtggagtctgggggaggcttggtcaaggctggagggtccctgagactctcctgtgtagcctctggattcaccttcagcgactactacatgtcctggattcgccaggctccagggaaggggctggagtggatttcatttattagtagtagtggtgataccatattttacgcagactctgtgaagggccgattcaccgtctccagggacagcgccaagaactcactgtatcttcaaatgaacagcctgaaagtcgaggacacggccgtgtattactgtgcgaggaagggggtgtccgacgaggaactactgcgcttctggggccagggaaccctggtcaccgtctcctca

**Heavy Chain Amino Acid Sequence:**

QVQLVESGGGLVKAGGSLRLSCVASGFTFSDYYMSWIRQAPGKGLEWISFISSSGDTIFYADSVKGRFTVSRDSAKNSLYLQMNSLKVEDTAVYYCARKGVSDEELLRFWGQGTLVTVSS

**Heavy Chain Nucleotide and Amino Acid Sequences:**

caggtgcagctggtggagtctgggggaggcttggtcaaggctggagggtccctgagactc

Q V Q L V E S G G G L V K A G G S L R L

tcctgtgtagcctctggattcaccttcagcgactactacatgtcctggattcgccaggct

S C V A S G F T F S D Y Y M S W I R Q A

ccagggaaggggctggagtggatttcatttattagtagtagtggtgataccatattttac

P G K G L E W I S F I S S S G D T I F Y

gcagactctgtgaagggccgattcaccgtctccagggacagcgccaagaactcactgtat

A D S V K G R F T V S R D S A K N S L Y

cttcaaatgaacagcctgaaagtcgaggacacggccgtgtattactgtgcgaggaagggg

L Q M N S L K V E D T A V Y Y C A R K G

gtgtccgacgaggaactactgcgcttctggggccagggaaccctggtcaccgtctcctca

V S D E E L L R F W G Q G T L V T V S S

**Light Chain Nucleotide Sequence:**

gatatcgtgctgactcaggacccctcggtgtcagtgtccccaggacaaacggccaggatcacctgctctggagatgcattgccaaaaaaatatgcttattggtaccagcagaagtcaggccaggcccctgtgctggtcatctatgaggacaccaaacgaccctccgggatccctgagagattctctggctccagctcagggacagtggccaccttgactatcagtggggcccaggtggaggatgaagctgactactattgttactcaacagacagcagcggtaatcagagggtattcggcggagggaccaagctgaccgtccta

**Light Chain Amino Acid Sequence:**

QVQLVESGGGLVKAGGSLRLSCVASGFTFSDYYMSWIRQAPGKGLEWISFISSSGDTIFYADSVKGRFTVSRDSAKNSLYLQMNSLKVEDTAVYYCARKGVSDEELLRFWGQGTLVTVSS

**Light Chain Nucleotide and Amino Acid Sequences:**

gatatcgtgctgactcaggacccctcggtgtcagtgtccccaggacaaacggccaggatc

D I V L T Q D P S V S V S P G Q T A R I

acctgctctggagatgcattgccaaaaaaatatgcttattggtaccagcagaagtcaggc

T C S G D A L P K K Y A Y W Y Q Q K S G

caggcccctgtgctggtcatctatgaggacaccaaacgaccctccgggatccctgagaga

Q A P V L V I Y E D T K R P S G I P E R

ttctctggctccagctcagggacagtggccaccttgactatcagtggggcccaggtggag

F S G S S S G T V A T L T I S G A Q V E

gatgaagctgactactattgttactcaacagacagcagcggtaatcagagggtattcggc

D E A D Y Y C Y S T D S S G N Q R V F G

ggagggaccaagctgaccgtccta

G G T K L T V L

**TRL1261**

**Heavy Chain Nucleotide Sequence:**

cagctggtggagtctgggggaggcgtggtgcagcctgggaggtccctgaggctctcctgtgcagcatctggactcaccttcagtgactatggcatgcactgggtccgccaggctccaggcaagggactggagtgggtggcagatatctggtatgacggaaatactaaatactatgcagactccgtggagggccgattcaccatctccagagacaattcccagagtacgctgtacctcgaaatgaacaacctgagggccgaagacacggctgtctactactgtgcgggggcctacagttacggctggttcgactactgggccagggaaccccggtcaccgtctcctcagc

**Heavy Chain Amino Acid Sequence:**

QLQLVESGGGVVQPGRSLRLSCAASGLTFSDYGMHWVRQAPGKGLEWVADIWYDGNTKYYADSVEGRFTISRDNSQSTLYLEMNNLRAEDTAVYYCAGAYSYGWFDYWAREPRSPSPQ

**Heavy Chain Nucleotide and Amino Acid Sequences:**

cagctgcagctggtggagtctgggggaggcgtggtgcagcctgggaggtccctgaggctc

Q L Q L V E S G G G V V Q P G R S L R L

tcctgtgcagcatctggactcaccttcagtgactatggcatgcactgggtccgccaggct

S C A A S G L T F S D Y G M H W V R Q A

ccaggcaagggactggagtgggtggcagatatctggtatgacggaaatactaaatactat

P G K G L E W V A D I W Y D G N T K Y Y

gcagactccgtggagggccgattcaccatctccagagacaattcccagagtacgctgtac

A D S V E G R F T I S R D N S Q S T L Y

ctcgaaatgaacaacctgagggccgaagacacggctgtctactactgtgcgggggcctac

L E M N N L R A E D T A V Y Y C A G A Y

agttacggctggttcgactactgggccagggaaccccggtcaccgtctcctcagc

S Y G W F D Y W A R E P R S P S P Q

**Light Chain Nucleotide Sequence:**

gatatcgtgatgacccagtctccagactccctggctgtgtctctgggcgagagggccaccatcaactgcaagtccagccagaacattttatacagctccaacaataagaactacttagcttggtaccaacagagaccaggacagtctcctaagctgctcttttactgggcatctacccgggcgtccggggtccctgaccgattcagtggcagcgggtctgggacagatttcactctcaccatcagcagcctgcagactgaagatgtggcagtttattactgtcagcaatattatactactccccccagcttcggccaagggacacggctggagataaga

**Light Chain Amino Acid Sequence:**

DIVMTQSPDSLAVSLGERATINCKSSQNILYSSNNKNYLAWYQQRPGQSPKLLFYWASTRASGVPDRFSGSGSGTDFTLTISSLQTEDVAVYYCQQYYTTPPSFGQGTRLEIR

**Light Chain Nucleotide and Amino Acid Sequences:**

gatatcgtgatgacccagtctccagactccctggctgtgtctctgggcgagagggccacc

D I V M T Q S P D S L A V S L G E R A T

atcaactgcaagtccagccagaacattttatacagctccaacaataagaactacttagct

I N C K S S Q N I L Y S S N N K N Y L A

tggtaccaacagagaccaggacagtctcctaagctgctcttttactgggcatctacccgg

W Y Q Q R P G Q S P K L L F Y W A S T R

gcgtccggggtccctgaccgattcagtggcagcgggtctgggacagatttcactctcacc

A S G V P D R F S G S G S G T D F T L T

atcagcagcctgcagactgaagatgtggcagtttattactgtcagcaatattatactact

I S S L Q T E D V A V Y Y C Q Q Y Y T T

ccccccagcttcggccaagggacacggctggagataaga

P P S F G Q G T R L E I R

**TRL1262**

**Heavy Chain Nucleotide Sequence:**

cagctgcagctggtggagtctgggggaggcgttgtccagcctgggaggtccctgagactctcctgtgcagcgtctggattaagtatcagcagttatggcatgcactgggtccgccaggctccaggcaagggactggaatgggtggcagttatatggtatgatggaactacaaaatactatgcagactccgtgaagggccgattcagcatctccagagacatttccaagaacacggtgtatctgcaaatgcagagcctgagagtcgaggacacggctgtgttttactgtgcgagtgctttcgcggaggggtacttggaacactggggccggggcaccctagtcaccgtctcctca

**Heavy Chain Amino Acid Sequence:**

QLQLVESGGGVVQPGRSLRLSCAASGLSISSYGMHWVRQAPGKGLEWVAVIWYDGTTKYYADSVKGRFSISRDISKNTVYLQMQSLRVEDTAVFYCASAFAEGYLEHWGRGTLVTVSS

**Heavy Chain Nucleotide and Amino Acid Sequences:**

cagctgcagctggtggagtctgggggaggcgttgtccagcctgggaggtccctgagactc

Q L Q L V E S G G G V V Q P G R S L R L

tcctgtgcagcgtctggattaagtatcagcagttatggcatgcactgggtccgccaggct

S C A A S G L S I S S Y G M H W V R Q A

ccaggcaagggactggaatgggtggcagttatatggtatgatggaactacaaaatactat

P G K G L E W V A V I W Y D G T T K Y Y

gcagactccgtgaagggccgattcagcatctccagagacatttccaagaacacggtgtat

A D S V K G R F S I S R D I S K N T V Y

ctgcaaatgcagagcctgagagtcgaggacacggctgtgttttactgtgcgagtgctttc

L Q M Q S L R V E D T A V F Y C A S A F

gcggaggggtacttggaacactggggccggggcaccctagtcaccgtctcctca

A E G Y L E H W G R G T L V T V S S

**Light Chain Nucleotide Sequence:**

gatatcgtgatgacccagtctccagactccctggctgtgtctctgggcgagagggccaccatcaactgcaagtccagccggagtattttagacagctccaaccataagaactacttaagttggtatcagcagaagccaggacagcctcctaaattactcactcgctgggcatctacccgggaatccggggtccctgaccgattcagtggcagcgggtctgggacagatttcactctcaccatcagcagcctgcaggctgaagatgtggcagtctattactgtcagcaatattttagtaccccggttacttttggccaggggaccaaggtggagatcaaa

**Light Chain Amino Acid Sequence:**

DIVMTQSPDSLAVSLGERATINCKSSRSILDSSNHKNYLSWYQQKPGQPPKLLTRWASTRESGVPDRFSGSGSGTDFTLTISSLQAEDVAVYYCQQYFSTPVTFGQGTKVEIK

**Light Chain Nucleotide and Amino Acid Sequences:**

gatatcgtgatgacccagtctccagactccctggctgtgtctctgggcgagagggccacc

D I V M T Q S P D S L A V S L G E R A T

atcaactgcaagtccagccggagtattttagacagctccaaccataagaactacttaagt

I N C K S S R S I L D S S N H K N Y L S

tggtatcagcagaagccaggacagcctcctaaattactcactcgctgggcatctacccgg

W Y Q Q K P G Q P P K L L T R W A S T R

gaatccggggtccctgaccgattcagtggcagcgggtctgggacagatttcactctcacc

E S G V P D R F S G S G S G T D F T L T

atcagcagcctgcaggctgaagatgtggcagtctattactgtcagcaatattttagtacc

I S S L Q A E D V A V Y Y C Q Q Y F S T

ccggttacttttggccaggggaccaaggtggagatcaaa

P V T F G Q G T K V E I K

**TRL1330**

**Heavy Chain Nucleotide Sequence:**

caggtgcagctggtggagtctggaactgaggtgaagaaccctggagcctcagtgaaggtctcctgcacggcctctggttacaaatttgacgaatatggtgtcagttgggtgcgacagtcccctggacaaggacttgagtggatgggatggatcagtgtttataatggcaagacaaactatagccagaactttcagggcagactcaccctgaccacagagacatccaccgacacagcctacatggagcttacgagcctcagacctgacgacacggccgtctattactgtgctacagacaaaaactggttcgacccctggggcccgggaaccctggtcaccgtctcctca

**Heavy Chain Amino Acid Sequence:**

QVQLVESGTEVKNPGASVKVSCTASGYKFDEYGVSWVRQSPGQGLEWMGWISVYNGKTNYSQNFQGRLTLTTETSTDTAYMELTSLRPDDTAVYYCATDKNWFDPWGPGTLVTVSS

**Heavy Chain Nucleotide and Amino Acid Sequences:**

caggtgcagctggtggagtctggaactgaggtgaagaaccctggagcctcagtgaaggtc

Q V Q L V E S G T E V K N P G A S V K V

tcctgcacggcctctggttacaaatttgacgaatatggtgtcagttgggtgcgacagtcc

S C T A S G Y K F D E Y G V S W V R Q S

cctggacaaggacttgagtggatgggatggatcagtgtttataatggcaagacaaactat

P G Q G L E W M G W I S V Y N G K T N Y

agccagaactttcagggcagactcaccctgaccacagagacatccaccgacacagcctac

S Q N F Q G R L T L T T E T S T D T A Y

atggagcttacgagcctcagacctgacgacacggccgtctattactgtgctacagacaaa

M E L T S L R P D D T A V Y Y C A T D K

aactggttcgacccctggggcccgggaaccctggtcaccgtctcctca

N W F D P W G P G T L V T V S S

**Light Chain Nucleotide Sequence:**

gatatcgtgttgactcagtctccctccgcgtccgggtctcctggacagtcaatcaccatctcctgcactggaaccaacactgattataattatgtttcctggtaccagcaccaccccggcaaagcccccaaagtcattatttatgacgtcaaaaagcggccctcgggggtccctagtcgcttctctggctccaggtctggcaacacggccaccctgaccgtctctgggctccagactgaggatgaggctgattattattgtgtctcatatgcagacaacaatcattatgtcttcggaagtgggaccaaggtcaccgtcctg

**Light Chain Amino Acid Sequence:**

DIVLTQSPSASGSPGQSITISCTGTNTDYNYVSWYQHHPGKAPKVIIYDVKKRPSGVPSRFSGSRSGNTATLTVSGLQTEDEADYYCVSYADNNHYVFGSGTKVTVL

**Light Chain Nucleotide and Amino Acid Sequences:**

gatatcgtgttgactcagtctccctccgcgtccgggtctcctggacagtcaatcaccatc

D I V L T Q S P S A S G S P G Q S I T I

tcctgcactggaaccaacactgattataattatgtttcctggtaccagcaccaccccggc

S C T G T N T D Y N Y V S W Y Q H H P G

aaagcccccaaagtcattatttatgacgtcaaaaagcggccctcgggggtccctagtcgc

K A P K V I I Y D V K K R P S G V P S R

ttctctggctccaggtctggcaacacggccaccctgaccgtctctgggctccagactgag

F S G S R S G N T A T L T V S G L Q T E

gatgaggctgattattattgtgtctcatatgcagacaacaatcattatgtcttcggaagt

D E A D Y Y C V S Y A D N N H Y V F G S

gggaccaaggtcaccgtcctg

G T K V T V L

**TRL1335**

**Heavy Chain Nucleotide Sequence:**

caggtgcagctggtggagtctggagcagaggtgaaaaagcccggggagtctctgaagatctcctgtaagggctctggatacaactttaccagttactggatcggctgggtgcgccagatgcccgggaaaggcctggagtggatgggagtcatctatcctgatgactctgataccagatacagcccgtcattcaaaggccaagtcaccatatcagccgacaagtccatcagcaccgccttcctgcagtggagcagtctaaaggcctcggacaccgccgtgtatcactgtgcgagacccccggactcctggggccagggaaccctggtcaccgtctcctca

**Heavy Chain Amino Acid Sequence:**

QVQLVESGAEVKKPGESLKISCKGSGYNFTSYWIGWVRQMPGKGLEWMGVIYPDDSDTRYSPSFKGQVTISADKSISTAFLQWSSLKASDTAVYHCARPPDSWGQGTLVTVSS

**Heavy Chain Nucleotide and Amino Acid Sequences:**

caggtgcagctggtggagtctggagcagaggtgaaaaagcccggggagtctctgaagatc

Q V Q L V E S G A E V K K P G E S L K I

tcctgtaagggctctggatacaactttaccagttactggatcggctgggtgcgccagatg

S C K G S G Y N F T S Y W I G W V R Q M

cccgggaaaggcctggagtggatgggagtcatctatcctgatgactctgataccagatac

P G K G L E W M G V I Y P D D S D T R Y

agcccgtcattcaaaggccaagtcaccatatcagccgacaagtccatcagcaccgccttc

S P S F K G Q V T I S A D K S I S T A F

ctgcagtggagcagtctaaaggcctcggacaccgccgtgtatcactgtgcgagacccccg

L Q W S S L K A S D T A V Y H C A R P P

gactcctggggccagggaaccctggtcaccgtctcctca

D S W G Q G T L V T V S S

**Light Chain Nucleotide Sequence:**

gatatcgtgatgacgcagtctccggccaccctgtctgtgtctccaggggaaagagccaccctctcctgcagggccagtcagagtgttagcagcaacttagcctggtaccagcagaaacctggcttggctcccagactcctcatcgtgggtgcatccaacagggccactggtatcccagccaggttcagtggcagtgggtctgggacagagttcactctcaccatcagcagcctgcagtctgaagattttgcattttattactgtcagcagtataataactggccattcactttcggccctgggaccaaagtggatgtcaaacga

**Light Chain Amino Acid Sequence:**

DIVMTQSPATLSVSPGERATLSCRASQSVSSNLAWYQQKPGLAPRLLIVGASNRATGIPARFSGSGSGTEFTLTISSLQSEDFAFYYCQQYNNWPFTFGPGTKVDVKR

**Light Chain Nucleotide and Amino Acid Sequences:**

gatatcgtgatgacgcagtctccggccaccctgtctgtgtctccaggggaaagagccacc

D I V M T Q S P A T L S V S P G E R A T

ctctcctgcagggccagtcagagtgttagcagcaacttagcctggtaccagcagaaacct

L S C R A S Q S V S S N L A W Y Q Q K P

ggcttggctcccagactcctcatcgtgggtgcatccaacagggccactggtatcccagcc

G L A P R L L I V G A S N R A T G I P A

aggttcagtggcagtgggtctgggacagagttcactctcaccatcagcagcctgcagtct

R F S G S G S G T E F T L T I S S L Q S

gaagattttgcattttattactgtcagcagtataataactggccattcactttcggccct

E D F A F Y Y C Q Q Y N N W P F T F G P

gggaccaaagtggatgtcaaacga

G T K V D V K R

**TRL1337**

**Heavy Chain Nucleotide Sequence:**

caggtgcagctgctcgagtcaggcccaggactggtgaagccttcggagaccccgtccctcacctgcactgtctctggtggctccatcaggagttactactggagctggatccggcagcccccagggaagggactggagtggattggatatatctattacagtgggagcaccaactacaacccctccctcaagagtcgagtcaccatatcagtagacatgtccaagaaccagttctccctgaagctgagctctgtgaccgccgcagacacggccatgtattactgtgcgagagtctacggaggttcggggagttacgactttgattactggggccagggaaccctggtcaccgtctcctca

**Heavy Chain Amino Acid Sequence:**

QVQLLESGPGLVKPSETPSLTCTVSGGSIRSYYWSWIRQPPGKGLEWIGYIYYSGSTNYNPSLKSRVTISVDMSKNQFSLKLSSVTAADTAMYYCARVYGGSGSYDFDYWGQGTLVTVSS

**Heavy Chain Nucleotide and Amino Acid Sequences:**

caggtgcagctgctcgagtcaggcccaggactggtgaagccttcggagaccccgtccctc

Q V Q L L E S G P G L V K P S E T P S L

acctgcactgtctctggtggctccatcaggagttactactggagctggatccggcagccc

T C T V S G G S I R S Y Y W S W I R Q P

ccagggaagggactggagtggattggatatatctattacagtgggagcaccaactacaac

P G K G L E W I G Y I Y Y S G S T N Y N

ccctccctcaagagtcgagtcaccatatcagtagacatgtccaagaaccagttctccctg

P S L K S R V T I S V D M S K N Q F S L

aagctgagctctgtgaccgccgcagacacggccatgtattactgtgcgagagtctacgga

K L S S V T A A D T A M Y Y C A R V Y G

ggttcggggagttacgactttgattactggggccagggaaccctggtcaccgtctcctca

G S G S Y D F D Y W G Q G T L V T V S S

**Light Chain Nucleotide Sequence:**

gatatcgtgttgacccagtctccctccgcgtccgggtctcctggacagtcagtcaccatctcctgcactggaaccagcagtgacgttggtggttataactatgtctcctggtaccaacagctcccaggcaaagcccccaaactcatgatttatgaggtcactaagcggccctcaggggtccctgatcgcttctctggctccaagtctggcaacacggcctccctgaccgtctctgggctccaggctgaggatgaggctgattattactgcagctcatttgcaggcagcaacaaccatgtggtattcggcggagggaccaagctgaccgtccta

**Light Chain Amino Acid Sequence:**

DIVLTQSPSASGSPGQSVTISCTGTSSDVGGYNYVSWYQQLPGKAPKLMIYEVTKRPSGVPDRFSGSKSGNTASLTVSGLQAEDEADYYCSSFAGSNNHVVFGGGTKLTVL

**Light Chain Nucleotide and Amino Acid Sequences:**

gatatcgtgttgacccagtctccctccgcgtccgggtctcctggacagtcagtcaccatc

D I V L T Q S P S A S G S P G Q S V T I

tcctgcactggaaccagcagtgacgttggtggttataactatgtctcctggtaccaacag

S C T G T S S D V G G Y N Y V S W Y Q Q

ctcccaggcaaagcccccaaactcatgatttatgaggtcactaagcggccctcaggggtc

L P G K A P K L M I Y E V T K R P S G V

cctgatcgcttctctggctccaagtctggcaacacggcctccctgaccgtctctgggctc

P D R F S G S K S G N T A S L T V S G L

caggctgaggatgaggctgattattactgcagctcatttgcaggcagcaacaaccatgtg

Q A E D E A D Y Y C S S F A G S N N H V

gtattcggcggagggaccaagctgaccgtccta

V F G G G T K L T V L

**TRL1338**

**Heavy Chain Nucleotide Sequence:**

caggtgcagctgaccttgagggagtctggtcctacgctggtgaaacccacacagaccctcacgctgacctgcaccttctctgggttctcactcagcactaatggagtgggtgtgggctggatccgtcagcccccaggaaaggccctggagtggcttgcaatcatttattgggatgatgataagcgctacagtccatctctgaaaagcaggctcaccatcaccaaggacacctccaaaaaccaggtggtccttacactgaccaacatggaccctgtggacacaggcacatattactgtgcacacattttaggcgcgtcgaattattggactggttatttgaggtactactttgactactggggccagggaaccctggtcaccgtctccaca

**Heavy Chain Amino Acid Sequence:**

QVQLTLRESGPTLVKPTQTLTLTCTFSGFSLSTNGVGVGWIRQPPGKALEWLAIIYWDDDKRYSPSLKSRLTITKDTSKNQVVLTLTNMDPVDTGTYYCAHILGASNYWTGYLRYYFDYWGQGTLVTVST

**Heavy Chain Nucleotide and Amino Acid Sequences:**

caggtgcagctgaccttgagggagtctggtcctacgctggtgaaacccacacagaccctc

Q V Q L T L R E S G P T L V K P T Q T L

acgctgacctgcaccttctctgggttctcactcagcactaatggagtgggtgtgggctgg

T L T C T F S G F S L S T N G V G V G W

atccgtcagcccccaggaaaggccctggagtggcttgcaatcatttattgggatgatgat

I R Q P P G K A L E W L A I I Y W D D D

aagcgctacagtccatctctgaaaagcaggctcaccatcaccaaggacacctccaaaaac

K R Y S P S L K S R L T I T K D T S K N

caggtggtccttacactgaccaacatggaccctgtggacacaggcacatattactgtgca

Q V V L T L T N M D P V D T G T Y Y C A

cacattttaggcgcgtcgaattattggactggttatttgaggtactactttgactactgg

H I L G A S N Y W T G Y L R Y Y F D Y W

ggccagggaaccctggtcaccgtctccaca

G Q G T L V T V S T

**Light Chain Nucleotide Sequence:**

gatatcgagatgacccagtctccctcggtgtcagtgtccccaggacagacggccaggatcacctgctctggagaaccattggcaaagcaatatgcttattggtatcagcagaagtcaggccaggcccctgtggtggtgatatataaagacactgagaggccctcagggatccctgagcgattctctggctccagctcagggacaacagtcacgttgaccatcagtggagtccaggcagaagacgaggctgactatcactgtgaatcaggagacagcagtggtacttatccggtattcggcggagggaccaagctgaccgtccta

**Light Chain Amino Acid Sequence:**

DIEMTQSPSVSVSPGQTARITCSGEPLAKQYAYWYQQKSGQAPVVVIYKDTERPSGIPERFSGSSSGTTVTLTISGVQAEDEADYHCESGDSSGTYPVFGGGTKLTVL

**Light Chain Nucleotide and Amino Acid Sequences:**

gatatcgagatgacccagtctccctcggtgtcagtgtccccaggacagacggccaggatc

D I E M T Q S P S V S V S P G Q T A R I

acctgctctggagaaccattggcaaagcaatatgcttattggtatcagcagaagtcaggc

T C S G E P L A K Q Y A Y W Y Q Q K S G

caggcccctgtggtggtgatatataaagacactgagaggccctcagggatccctgagcga

Q A P V V V I Y K D T E R P S G I P E R

ttctctggctccagctcagggacaacagtcacgttgaccatcagtggagtccaggcagaa

F S G S S S G T T V T L T I S G V Q A E

gacgaggctgactatcactgtgaatcaggagacagcagtggtacttatccggtattcggc

D E A D Y H C E S G D S S G T Y P V F G

ggagggaccaagctgaccgtccta

G G T K L T V L

**TRL1339**

**Heavy Chain Nucleotide Sequence:**

cagctgcagctggtggagtctggagcagaggtgaaaaagccaggggagtctctgaagatctcctgtcagggatctggatacagctttagaaactactggatcggctgggtgcgccagaggcccggggaaggcctggagtggatggggatcatctatcccggtgactctgaaaccagatacggcccgtccttcagaggccaggtcaccatctcagccgacaagtccatcagcaccgcctacctgcagtggagtagcctgaaggcctcggacaccgccatgtattactgtgcgagggttggatattgtagtagtggcagctgctttctaggctggttcgacccctggggccagggaaccctggtcaccgtctcctca

**Heavy Chain Amino Acid Sequence:**

QLQLVESGAEVKKPGESLKISCQGSGYSFRNYWIGWVRQRPGEGLEWMGIIYPGDSETRYGPSFRGQVTISADKSISTAYLQWSSLKASDTAMYYCARVGYCSSGSCFLGWFDPWGQGTLVTVSS

**Heavy Chain Nucleotide and Amino Acid Sequences:**

cagctgcagctggtggagtctggagcagaggtgaaaaagccaggggagtctctgaagatc

Q L Q L V E S G A E V K K P G E S L K I

tcctgtcagggatctggatacagctttagaaactactggatcggctgggtgcgccagagg

S C Q G S G Y S F R N Y W I G W V R Q R

cccggggaaggcctggagtggatggggatcatctatcccggtgactctgaaaccagatac

P G E G L E W M G I I Y P G D S E T R Y

ggcccgtccttcagaggccaggtcaccatctcagccgacaagtccatcagcaccgcctac

G P S F R G Q V T I S A D K S I S T A Y

ctgcagtggagtagcctgaaggcctcggacaccgccatgtattactgtgcgagggttgga

L Q W S S L K A S D T A M Y Y C A R V G

tattgtagtagtggcagctgctttctaggctggttcgacccctggggccagggaaccctg

Y C S S G S C F L G W F D P W G Q G T L

gtcaccgtctcctca

V T V S S

**Light Chain Nucleotide Sequence:**

gatatcgtgatgactcagtctccagactccctggctgtgtctctgggcgagagggccaccatcaactgcaagtccagccagagtattttacacagctccaacaataagaactacttagcttggtaccagcagaaaccaagacagcctcctaggctgctcatttactgggcatctacccgggaatccggggtccctgaccgattcagtggcagcgggtctgggacagatttcactctcaccatcagcagcctgcaggctgaagatgtggcactttattactgtcagcaatattataatagtcctcaaacttttggccaggggaccaagctggagatcaaa

**Light Chain Amino Acid Sequence:**

DIVMTQSPDSLAVSLGERATINCKSSQSILHSSNNKNYLAWYQQKPRQPPRLLIYWASTRESGVPDRFSGSGSGTDFTLTISSLQAEDVALYYCQQYYNSPQTFGQGTKLEIK

**Light Chain Nucleotide and Amino Acid Sequences:**

gatatcgtgatgactcagtctccagactccctggctgtgtctctgggcgagagggccacc

D I V M T Q S P D S L A V S L G E R A T

atcaactgcaagtccagccagagtattttacacagctccaacaataagaactacttagct

I N C K S S Q S I L H S S N N K N Y L A

tggtaccagcagaaaccaagacagcctcctaggctgctcatttactgggcatctacccgg

W Y Q Q K P R Q P P R L L I Y W A S T R

gaatccggggtccctgaccgattcagtggcagcgggtctgggacagatttcactctcacc

E S G V P D R F S G S G S G T D F T L T

atcagcagcctgcaggctgaagatgtggcactttattactgtcagcaatattataatagt

I S S L Q A E D V A L Y Y C Q Q Y Y N S

cctcaaacttttggccaggggaccaagctggagatcaaa

P Q T F G Q G T K L E I K

**TRL1341**

**Heavy Chain Nucleotide Sequence:**

caggtgcagctgcaggagtcggggggaggcttggtccagcctggggggtccctgaaactctcctgtgcagcctctgggttcatcttcagtggctctactatgcactgggtccgccaggcttccgggaaagggctggagtgggttggccgtatcagaagcaaaactaacaattacgcgacagcatatgctgcgtcggtgaaaggcaggttcaccatctccagagatgattcaaagaacacggcgtatctgcaaatgaacagcctgaaaaccgaggacacggccgtgtattattgtattagcttacctggcgggtatagcagtggacagggaaccctggtcaccgtctcctca

**Heavy Chain Amino Acid Sequence:**

QVQLQESGGGLVQPGGSLKLSCAASGFIFSGSTMHWVRQASGKGLEWVGRIRSKTNNYATAYAASVKGRFTISRDDSKNTAYLQMNSLKTEDTAVYYCISLPGGYSSGQGTLVTVSS

**Heavy Chain Nucleotide and Amino Acid Sequences:**

caggtgcagctgcaggagtcggggggaggcttggtccagcctggggggtccctgaaactc

Q V Q L Q E S G G G L V Q P G G S L K L

tcctgtgcagcctctgggttcatcttcagtggctctactatgcactgggtccgccaggct

S C A A S G F I F S G S T M H W V R Q A

tccgggaaagggctggagtgggttggccgtatcagaagcaaaactaacaattacgcgaca

S G K G L E W V G R I R S K T N N Y A T

gcatatgctgcgtcggtgaaaggcaggttcaccatctccagagatgattcaaagaacacg

A Y A A S V K G R F T I S R D D S K N T

gcgtatctgcaaatgaacagcctgaaaaccgaggacacggccgtgtattattgtattagc

A Y L Q M N S L K T E D T A V Y Y C I S

ttacctggcgggtatagcagtggacagggaaccctggtcaccgtctcctca

L P G G Y S S G Q G T L V T V S S

**Light Chain Nucleotide Sequence:**

gatatcatgctgactcagccaccctcggtgtcagtgtccccaggacaaacggccaggatcacctgctctggagatgcattgccaaaaaaatatacttattggtatcagcagaagtcaggccaggcccctgttttggtcatctatgaggacagcaaacgaccctccgagatccctgagagattctctgccttcacctcatggacgacggccaccttgactataactggggcccaggtgggagatgaagctgactactactgttattcaacagacatcactggtgatataggagtgttcggcggagggaccaagctgaccgtcctg

**Light Chain Amino Acid Sequence:**

DIMLTQPPSVSVSPGQTARITCSGDALPKKYTYWYQQKSGQAPVLVIYEDSKRPSEIPERFSAFTSWTTATLTITGAQVGDEADYYCYSTDITGDIGVFGGGTKLTVL

**Light Chain Nucleotide and Amino Acid Sequences:**

gatatcatgctgactcagccaccctcggtgtcagtgtccccaggacaaacggccaggatc

D I M L T Q P P S V S V S P G Q T A R I

acctgctctggagatgcattgccaaaaaaatatacttattggtatcagcagaagtcaggc

T C S G D A L P K K Y T Y W Y Q Q K S G

caggcccctgttttggtcatctatgaggacagcaaacgaccctccgagatccctgagaga

Q A P V L V I Y E D S K R P S E I P E R

ttctctgccttcacctcatggacgacggccaccttgactataactggggcccaggtggga

F S A F T S W T T A T L T I T G A Q V G

gatgaagctgactactactgttattcaacagacatcactggtgatataggagtgttcggc

D E A D Y Y C Y S T D I T G D I G V F G

ggagggaccaagctgaccgtcctg

G G T K L T V L

**TRL1347**

**Heavy Chain Nucleotide Sequence:**

caggtgcagctggtgcagtctgggggaggcttggtccagcctggggggtccctgaaagtctcctgtgtaggctctggattcacgttcagtgcctctactatacactgggtccgccaggcctccgggaaagggctggagtgggttggccgtatcagaagcaaagctaacaattacgcgacagtatctgctgcgtcgctgaaaggcaggttcaccatctccagagatgattcaaagaacacggcgtatctccaagtgaacagcctgaaaatcgaggacacggccatttattactgtactagacctacggcctgcggtgaccgcgtctgctggcacggggcttggggccagggaacccaggtcaccgtctcccca

**Heavy Chain Amino Acid Sequence:**

QVQLVQSGGGLVQPGGSLKVSCVGSGFTFSASTIHWVRQASGKGLEWVGRIRSKANNYATVSAASLKGRFTISRDDSKNTAYLQVNSLKIEDTAIYYCTRPTACGDRVCWHGAWGQGTQVTVSP

**Heavy Chain Nucleotide and Amino Acid Sequences:**

caggtgcagctggtgcagtctgggggaggcttggtccagcctggggggtccctgaaagtc

Q V Q L V Q S G G G L V Q P G G S L K V

tcctgtgtaggctctggattcacgttcagtgcctctactatacactgggtccgccaggcc

S C V G S G F T F S A S T I H W V R Q A

tccgggaaagggctggagtgggttggccgtatcagaagcaaagctaacaattacgcgaca

S G K G L E W V G R I R S K A N N Y A T

gtatctgctgcgtcgctgaaaggcaggttcaccatctccagagatgattcaaagaacacg

V S A A S L K G R F T I S R D D S K N T

gcgtatctccaagtgaacagcctgaaaatcgaggacacggccatttattactgtactaga

A Y L Q V N S L K I E D T A I Y Y C T R

cctacggcctgcggtgaccgcgtctgctggcacggggcttggggccagggaacccaggtc

P T A C G D R V C W H G A W G Q G T Q V

accgtctcccca

T V S P

**Light Chain Nucleotide Sequence:**

gatatcgtgctgacgcagtctccctcagcgtctgggacccccgggcagagggtcaccatctcttgttctggaagcagatccaacctcggaaataataatgtcaactggtaccagcagctcccgggaacggcccccaaactcctcatctttgataataatgagaggccctcaggggtcccaggccgattctctggctccaagtctggcacctcagcctccctggccatcagtgggctccggtctgaggatgaggctgattattactgtgcatcatgggatgacagcctgaatggttgggtgttcggcggagggaccaaggtgaccgtcctg

**Light Chain Amino Acid Sequence:**

DIVLTQSPSASGTPGQRVTISCSGSRSNLGNNNVNWYQQLPGTAPKLLIFDNNERPSGVPGRFSGSKSGTSASLAISGLRSEDEADYYCASWDDSLNGWVFGGGTKVTVL

**Light Chain Nucleotide and Amino Acid Sequences:**

gatatcgtgctgacgcagtctccctcagcgtctgggacccccgggcagagggtcaccatc

D I V L T Q S P S A S G T P G Q R V T I

tcttgttctggaagcagatccaacctcggaaataataatgtcaactggtaccagcagctc

S C S G S R S N L G N N N V N W Y Q Q L

ccgggaacggcccccaaactcctcatctttgataataatgagaggccctcaggggtccca

P G T A P K L L I F D N N E R P S G V P

ggccgattctctggctccaagtctggcacctcagcctccctggccatcagtgggctccgg

G R F S G S K S G T S A S L A I S G L R

tctgaggatgaggctgattattactgtgcatcatgggatgacagcctgaatggttgggtg

S E D E A D Y Y C A S W D D S L N G W V

ttcggcggagggaccaaggtgaccgtcctg

F G G G T K V T V L

**TRL1361**

**Heavy Chain Nucleotide Sequence:**

caggtgcagctggtggagtctgggggaggcttggcacagcctggggggtccctgagactctcctgtgcagcctctggattcatctttaacacctatgccatgggctgggtccgccaggctccagggaaggggctggagtgggtctcaactgttagtgctcctggtgctggcacatactacacagactccgtgaagggccgattcatcatctccagagacaattccaagaacatactgtatctgcaaatgaacaggctgagagtcgaggacacggccgtctattactgtgcgagggatcagggggggccagcagtggctggtgcaaggatctttgactactggggccagggagccctggtcaccgtctcctca

**Heavy Chain Amino Acid Sequence:**

QVQLVESGGGLAQPGGSLRLSCAASGFIFNTYAMGWVRQAPGKGLEWVSTVSAPGAGTYYTDSVKGRFIISRDNSKNILYLQMNRLRVEDTAVYYCARDQGGPAVAGARIFDYWGQGALVTVSS

**Heavy Chain Nucleotide and Amino Acid Sequences:**

caggtgcagctggtggagtctgggggaggcttggcacagcctggggggtccctgagactc

Q V Q L V E S G G G L A Q P G G S L R L

tcctgtgcagcctctggattcatctttaacacctatgccatgggctgggtccgccaggct

S C A A S G F I F N T Y A M G W V R Q A

ccagggaaggggctggagtgggtctcaactgttagtgctcctggtgctggcacatactac

P G K G L E W V S T V S A P G A G T Y Y

acagactccgtgaagggccgattcatcatctccagagacaattccaagaacatactgtat

T D S V K G R F I I S R D N S K N I L Y

ctgcaaatgaacaggctgagagtcgaggacacggccgtctattactgtgcgagggatcag

L Q M N R L R V E D T A V Y Y C A R D Q

ggggggccagcagtggctggtgcaaggatctttgactactggggccagggagccctggtc

G G P A V A G A R I F D Y W G Q G A L V

accgtctcctca

T V S S

**Light Chain Nucleotide Sequence:**

gatatcgtgttgactcagtctccactctctctgtccgtcacccctggacagccggcctccatctcctgcaagtctagtcagagcctcctgcgtagtgatggaaaaacatatttgtgctggtacctgcagaagccaggccagcctccacagctcctgatctatgaagtttccaaccgggtctctggagtgccagacaggttcagtggcagcgggtcagggacagatttcacactgaaaatcagccgggtggaggctgaggatgttggggtttattactgcatgcaaagtatacagcttcggacgttcggccaagggaccaaggtggaaatcaaacgaa

**Light Chain Amino Acid Sequence:**

DIVLTQSPLSLSVTPGQPASISCKSSQSLLRSDGKTYLCWYLQKPGQPPQLLIYEVSNRVSGVPDRFSGSGSGTDFTLKISRVEAEDVGVYYCMQSIQLRTFGQGTKVEIKR

**Light Chain Nucleotide and Amino Acid Sequences:**

gatatcgtgttgactcagtctccactctctctgtccgtcacccctggacagccggcctcc

D I V L T Q S P L S L S V T P G Q P A S

atctcctgcaagtctagtcagagcctcctgcgtagtgatggaaaaacatatttgtgctgg

I S C K S S Q S L L R S D G K T Y L C W

tacctgcagaagccaggccagcctccacagctcctgatctatgaagtttccaaccgggtc

Y L Q K P G Q P P Q L L I Y E V S N R V

tctggagtgccagacaggttcagtggcagcgggtcagggacagatttcacactgaaaatc

S G V P D R F S G S G S G T D F T L K I

agccgggtggaggctgaggatgttggggtttattactgcatgcaaagtatacagcttcgg

S R V E A E D V G V Y Y C M Q S I Q L R

acgttcggccaagggaccaaggtggaaatcaaacgaa

T F G Q G T K V E I K R

**Supplemental Information 2: ELISA binding data supporting the Kd calculation presented in Table 2 are provided here, including duplicate assays.**

| HU Sa  **(2ug/ml)** |  |  |  |  |  |  |  |  |  |  |  |  |
| --- | --- | --- | --- | --- | --- | --- | --- | --- | --- | --- | --- | --- |
| mAb | nM | | | | | | | | | | | |
|  | 600.000 | 200.000 | 66.667 | 22.222 | 7.407 | 2.469 | 0.823 | 0.274 | 0.091 | 0.030 | 0.010 | BSA |
| 1335 | 1.5317 | 0.6572 | 0.2635 | 0.1507 | 0.1189 | 0.1241 | 0.3002 | 0.8542 | 2.1619 | 0.2930 | 0.4104 | 0.2344 |
|  | 1.5540 | 0.8499 | 0.3747 | 0.1886 | 0.1599 | 0.1274 | 0.1306 | 0.1622 | 0.1664 | 0.1524 | 0.1579 | 0.1814 |
| 1337 | 3.4717 | 3.5068 | 3.2387 | 2.9420 | 2.1356 | 1.0871 | 0.4609 | 0.2464 | 0.1670 | 0.1518 | 0.1748 | 0.1767 |
|  | 0.0466 | 0.0408 | 0.0454 | 0.0459 | 0.0553 | 0.0501 | 0.0479 | 0.0537 | 0.0537 | 0.0480 | 0.0487 | 0.0487 |
| 1338 | 3.1424 | 3.0743 | 2.5049 | 1.4757 | 0.6188 | 0.3057 | 0.2226 | 0.1395 | 0.1597 | 0.1479 | 0.1526 | 0.1708 |
|  | 3.1034 | 3.0546 | 2.4796 | 1.4779 | 0.6236 | 0.2822 | 0.1748 | 0.1380 | 0.1225 | 0.1334 | 0.1392 | 0.1882 |
| 1341 | 0.5630 | 0.3680 | 0.1828 | 0.3354 | 0.1108 | 0.1053 | 2.3041 | 0.1594 | 0.5242 | 1.8906 | 1.7213 | 2.1718 |
|  | 0.2991 | 0.1917 | 0.1526 | 0.1034 | 0.1185 | 0.1097 | 0.1355 | 0.1203 | 0.1894 | 0.3087 | 0.1969 | 0.1734 |
| 1347 | 3.0588 | 1.5633 | 0.7104 | 0.3633 | 0.1700 | 0.1502 | 0.1215 | 1.1993 | 1.0513 | 0.2640 | 0.2315 | 0.1965 |
|  | 3.0142 | 1.7538 | 0.8667 | 0.4047 | 0.1907 | 0.1415 | 0.1309 | 0.3216 | 0.1484 | 0.1311 | 0.1384 | 0.1559 |
| 1361 | 3.0809 | 3.2409 | 2.8670 | 2.5478 | 1.6935 | 0.9592 | 0.4065 | 0.2942 | 0.1516 | 0.1441 | 0.1419 | 0.1841 |
|  | 3.1220 | 3.1725 | 2.9109 | 2.5898 | 1.8792 | 1.1276 | 0.4122 | 0.3008 | 0.1583 | 0.1480 | 0.1630 | 0.1646 |
|  |  |  |  |  |  |  |  |  |  |  |  |  |
| mAb | nM | | | | | | | | | | | |
|  | 60.000 | 20.000 | 6.667 | 2.222 | 0.741 | 0.247 | 0.082 | 0.027 | 0.009 | 0.003 | 0.001 | BSA |
| 1068 | 4.0000 | 3.5945 | 3.2777 | 3.3909 | 3.2463 | 3.0740 | 2.8504 | 2.3692 | 1.4955 | 0.7997 | 0.4282 | 0.1431 |
|  | 2.3824 | 3.3306 | 3.4601 | 3.3168 | 3.2956 | 3.1382 | 2.8673 | 2.4312 | 1.4636 | 0.7675 | 0.4129 | 0.1586 |
|  |  |  |  |  |  |  |  |  |  |  |  |  |
| mAb | nM | | | | | | | | | | | |
|  | 20.000 | 6.667 | 2.222 | 0.741 | 0.247 | 0.082 | 0.027 | 0.009 | 0.003 | 0.001 | 0.000 | BSA |
| 1330 | 2.9676 | 3.2357 | 3.0738 | 2.8718 | 2.5772 | 2.0805 | 1.0262 | 0.5564 | 0.2689 | 0.1982 | 0.1714 | 0.1210 |
|  | 3.1605 | 3.2530 | 3.0347 | 2.8284 | 2.4772 | 1.7620 | 0.8822 | 0.4061 | 0.2223 | 0.1488 | 0.1818 | 0.3704 |

| IHF Ab  **(2ug/ml)** | |  |  |  |  |  |  |  |  |  |  |  |
| --- | --- | --- | --- | --- | --- | --- | --- | --- | --- | --- | --- | --- |
| mAb | nM | | | | | | | | | | | |
|  | 600.000 | 200.000 | 66.667 | 22.222 | 7.407 | 2.469 | 0.823 | 0.274 | 0.091 | 0.030 | 0.010 | BSA |
| 1335 | 0.2469 | 0.1880 | 0.1256 | 0.1483 | 0.1110 | 0.1364 | 0.2352 | 0.1755 | 0.1655 | 0.1924 | 0.2544 | 0.2531 |
|  | 0.2147 | 0.1463 | 0.1248 | 0.1234 | 0.1140 | 0.1296 | 0.1384 | 0.1343 | 0.1452 | 0.1895 | 0.1998 | 0.2451 |
| 1337 | 0.1327 | 0.0917 | 0.0900 | 0.0957 | 0.1083 | 0.1210 | 0.1360 | 0.5302 | 0.1356 | 0.1711 | 0.2095 | 0.2242 |
|  | 0.1273 | 0.1373 | 0.1847 | 0.1645 | 0.1143 | 0.1399 | 0.2034 | 0.1490 | 0.1574 | 0.1870 | 0.2152 | 0.2051 |
| 1338 | 0.2568 | 0.1342 | 0.1224 | 0.1570 | 0.1049 | 0.1215 | 0.1528 | 0.1748 | 0.2098 | 0.1570 | 0.1960 | 0.1907 |
|  | 0.8386 | 0.1200 | 0.1163 | 0.1097 | 0.1116 | 0.1170 | 0.1313 | 0.1245 | 0.1442 | 0.2102 | 0.1762 | 0.2086 |
| 1341 | 0.2631 | 0.1470 | 0.1060 | 0.0993 | 0.1066 | 0.1206 | 0.1241 | 0.1347 | 0.1536 | 0.1400 | 0.1749 | 0.1827 |
|  | 0.9040 | 0.1674 | 0.1725 | 0.1216 | 0.1209 | 0.1629 | 0.2583 | 0.1314 | 0.1415 | 0.1763 | 0.2654 | 0.2050 |
| 1347 | 0.3392 | 0.3035 | 0.2367 | 0.1388 | 0.1430 | 0.1659 | 0.1640 | 0.1711 | 0.1776 | 0.2209 | 0.2926 | 0.3083 |
|  | 0.3560 | 0.2640 | 0.2352 | 0.1264 | 0.1260 | 0.1832 | 0.2143 | 0.2083 | 0.1550 | 0.1784 | 0.2468 | 0.3011 |
| 1361 | 0.2692 | 0.2412 | 0.1736 | 0.1557 | 0.1712 | 0.1664 | 0.1766 | 0.1732 | 0.1594 | 0.1906 | 0.2366 | 0.2601 |
|  | 0.3787 | 0.2401 | 0.2239 | 0.1273 | 0.1485 | 0.1457 | 0.1736 | 0.1541 | 0.1687 | 0.1899 | 0.2028 | 0.2153 |
|  |  |  |  |  |  |  |  |  |  |  |  |  |
| mAb | nM | | | | | | | | | | | |
|  | 60.000 | 20.000 | 6.667 | 2.222 | 0.741 | 0.247 | 0.082 | 0.027 | 0.009 | 0.003 | 0.001 | BSA |
| 1068 | 3.5869 | 3.6927 | 3.6204 | 3.5648 | 3.4531 | 3.3907 | 3.2175 | 3.0193 | 2.2404 | 1.4969 | 0.7042 | 0.1948 |
|  | 3.6584 | 3.7184 | 3.6870 | 3.6537 | 3.5573 | 3.4383 | 3.2892 | 2.8528 | 2.1319 | 1.3417 | 0.9232 | 0.2179 |
|  |  |  |  |  |  |  |  |  |  |  |  |  |
| mAb | nM | | | | | | | | | | | |
|  | 20.000 | 6.667 | 2.222 | 0.741 | 0.247 | 0.082 | 0.027 | 0.009 | 0.003 | 0.001 | 0.000 | BSA |
| 1330 | 3.4700 | 3.5116 | 3.3273 | 3.1516 | 2.8238 | 2.1801 | 1.2048 | 0.6096 | 0.3723 | 0.2476 | 0.2282 | 0.2082 |
|  | 3.3907 | 3.4320 | 3.2685 | 3.0752 | 2.7431 | 2.1154 | 1.1244 | 0.5485 | 0.2737 | 0.2331 | 0.2906 | 0.2273 |

| IHF Kp  **(2ug/ml)** | |  |  |  |  |  |  |  |  |  |  |  |
| --- | --- | --- | --- | --- | --- | --- | --- | --- | --- | --- | --- | --- |
| mAb | nM | | | | | | | | | | | |
|  | 600.000 | 200.000 | 66.667 | 22.222 | 7.407 | 2.469 | 0.823 | 0.274 | 0.091 | 0.030 | 0.010 | BSA |
| 1335 | 0.2266 | 0.1432 | 0.2501 | 0.1246 | 0.1115 | 0.1361 | 0.1426 | 0.1723 | 0.1970 | 0.2297 | 0.2423 | 0.2836 |
|  | 0.2148 | 0.1158 | 0.1187 | 0.1052 | 0.1217 | 0.1312 | 0.1426 | 0.2039 | 0.1799 | 0.1824 | 0.2457 | 0.4021 |
| 1337 | 0.0960 | 0.0901 | 0.1070 | 0.0970 | 0.1184 | 0.1066 | 0.1365 | 0.1366 | 0.1512 | 0.1719 | 0.2007 | 0.2485 |
|  | 0.1034 | 0.1200 | 0.1070 | 0.0937 | 0.1050 | 0.1119 | 0.1398 | 0.1428 | 0.1611 | 0.1884 | 0.2095 | 0.3012 |
| 1338 | 0.1847 | 0.1119 | 0.1043 | 0.1239 | 0.1299 | 0.1207 | 0.1444 | 0.1574 | 0.1475 | 0.1784 | 0.2952 | 0.2471 |
|  | 0.3469 | 0.1440 | 0.0998 | 0.1002 | 0.1143 | 0.1117 | 0.1260 | 0.1384 | 0.1526 | 0.1779 | 0.2425 | 0.2565 |
| 1341 | 0.3794 | 0.1688 | 0.1307 | 0.1106 | 0.1150 | 0.1534 | 0.1234 | 0.1375 | 0.1773 | 0.2514 | 0.2446 | 0.3091 |
|  | 0.2606 | 0.1381 | 0.1111 | 0.1029 | 0.1288 | 0.1223 | 0.1338 | 0.1236 | 0.1425 | 0.1654 | 0.3228 | 0.1955 |
| 1347 | 0.2385 | 0.1653 | 0.1656 | 0.1785 | 0.1543 | 0.1758 | 0.1949 | 0.2256 | 0.2073 | 0.2660 | 0.2559 | 0.3019 |
|  | 0.4145 | 0.2399 | 0.1441 | 0.1293 | 0.1571 | 0.1639 | 0.1697 | 0.1892 | 0.1886 | 0.2109 | 0.2469 | 0.3131 |
| 1361 | 0.3264 | 0.2003 | 0.2949 | 0.1399 | 0.1618 | 0.4506 | 0.7933 | 0.1719 | 0.1693 | 0.1946 | 0.2306 | 0.2732 |
|  | 0.3371 | 0.1782 | 0.1557 | 0.1247 | 0.1380 | 0.1400 | 0.1597 | 0.5720 | 0.1870 | 0.2057 | 0.2234 | 0.2767 |
|  |  |  |  |  |  |  |  |  |  |  |  |  |
| mAb | nM | | | | | | | | | | | |
|  | 60.000 | 20.000 | 6.667 | 2.222 | 0.741 | 0.247 | 0.082 | 0.027 | 0.009 | 0.003 | 0.001 | BSA |
| 1068 | 3.6348 | 3.7382 | 3.6920 | 3.6406 | 3.4822 | 3.3173 | 2.9972 | 2.3434 | 1.2699 | 0.6404 | 0.3775 | 0.2746 |
|  | 3.7920 | 3.8438 | 3.8127 | 3.7826 | 3.5583 | 3.3468 | 3.0816 | 2.3423 | 1.2774 | 0.6065 | 0.3624 | 0.2898 |
|  |  |  |  |  |  |  |  |  |  |  |  |  |
| mAb | nM | | | | | | | | | | | |
|  | 20.000 | 6.667 | 2.222 | 0.741 | 0.247 | 0.082 | 0.027 | 0.009 | 0.003 | 0.001 | 0.000 | BSA |
| 1330 (sup) | 3.3909 | 3.5504 | 3.3416 | 3.2344 | 2.8461 | 2.0747 | 1.0951 | 0.5450 | 0.3284 | 0.2539 | 0.2325 | 0.2760 |
|  | 3.5082 | 3.5940 | 3.3910 | 3.2003 | 2.8566 | 2.1576 | 1.1518 | 0.5142 | 0.3370 | 0.2668 | 0.3181 | 0.2980 |

| IHF Hi  **(2ug/ml)** | |  |  |  |  |  |  |  |  |  |  |  |
| --- | --- | --- | --- | --- | --- | --- | --- | --- | --- | --- | --- | --- |
| mAb | nM | | | | | | | | | | | |
|  | 600.000 | 200.000 | 66.667 | 22.222 | 7.407 | 2.469 | 0.823 | 0.274 | 0.091 | 0.030 | 0.010 | BSA |
| 1335 | 0.3091 | 0.1915 | 0.2812 | 0.2363 | 0.1815 | 0.3653 | 0.4215 | 0.2182 | 0.2423 | 0.309 | 0.2875 | 0.2837 |
|  | 0.1911 | 0.13 | 0.1302 | 0.1279 | 0.1408 | 0.1784 | 0.6762 | 0.1924 | 0.1792 | 0.2142 | 0.2602 | 0.3572 |
| 1337 | 0.158 | 0.1167 | 0.1158 | 0.1248 | 0.1842 | 0.2281 | 0.7298 | 0.2846 | 0.1998 | 0.2825 | 0.2571 | 0.2902 |
|  | 0.0486 | 0.1147 | 0.1202 | 0.1144 | 0.1357 | 0.1476 | 0.1585 | 0.1773 | 0.1893 | 0.2003 | 0.2079 | 0.2438 |
| 1338 | 0.2751 | 0.1607 | 0.1418 | 0.1316 | 0.1601 | 0.1509 | 0.1811 | 0.1781 | 0.1959 | 0.2004 | 0.2294 | 0.2437 |
|  | 0.2169 | 0.1338 | 0.1316 | 0.1494 | 0.1263 | 0.1337 | 0.1496 | 0.1487 | 0.1945 | 0.1881 | 0.2138 | 0.2742 |
| 1341 | 0.2325 | 0.1491 | 0.1232 | 0.1626 | 0.1738 | 0.1369 | 0.1431 | 0.187 | 0.2245 | 0.1907 | 0.2002 | 0.2312 |
|  | 0.2394 | 0.2239 | 0.2023 | 0.1719 | 0.1958 | 0.221 | 0.2542 | 0.2107 | 0.2209 | 0.2367 | 0.3602 | 0.2787 |
| 1347 | 0.1104 | 0.0992 | 0.113 | 0.1285 | 0.1283 | 0.1431 | 0.1464 | 0.1853 | 0.1707 | 0.2177 | 0.2945 | 0.3064 |
|  | 0.096 | 0.1805 | 0.1036 | 0.1005 | 0.1234 | 0.1433 | 0.1462 | 0.1578 | 0.1576 | 0.191 | 0.221 | 0.3284 |
| 1361 | 0.2187 | 0.1647 | 0.111 | 0.1096 | 0.1168 | 0.247 | 0.1777 | 0.1601 | 0.1686 | 0.2016 | 0.2392 | 0.2867 |
|  | 0.2243 | 0.1645 | 0.1901 | 0.1313 | 0.1911 | 0.2008 | 0.1552 | 0.1799 | 0.1771 | 0.2102 | 0.2332 | 0.2678 |
|  |  |  |  |  |  |  |  |  |  |  |  |  |
| mAb | nM | | | | | | | | | | | |
|  | 60.000 | 20.000 | 6.667 | 2.222 | 0.741 | 0.247 | 0.082 | 0.027 | 0.009 | 0.003 | 0.001 | BSA |
| 1068 | 2.0181 | 1.3489 | 0.5185 | 0.2725 | 0.171 | 0.143 | 0.1618 | 0.1518 | 0.1638 | 0.1987 | 0.21 | 0.2283 |
|  | 2.3461 | 1.3935 | 1.0526 | 0.3626 | 0.1902 | 0.1454 | 0.1428 | 0.1257 | 0.194 | 0.181 | 0.1816 | 0.2367 |
|  |  |  |  |  |  |  |  |  |  |  |  |  |
| mAb | nM | | | | | | | | | | | |
|  | 20.000 | 6.667 | 2.222 | 0.741 | 0.247 | 0.082 | 0.027 | 0.009 | 0.003 | 0.001 | 0.000 | BSA |
| 1330 | 3.4075 | 3.4685 | 3.2913 | 3.0877 | 2.731 | 1.9432 | 1.0622 | 0.5576 | 0.3274 | 0.4356 | 0.2787 | 0.3064 |
|  | 3.4714 | 3.442 | 3.2955 | 3.1107 | 2.764 | 1.9691 | 1.1157 | 0.5352 | 0.3498 | 0.2689 | 0.4117 | 0.3929 |
